# Comparative outcomes of advanced medical rescue therapies in steroid-refractory acute severe ulcerative colitis: a systematic review and network meta-analysis

**DOI:** 10.3389/fgstr.2026.1937925

**Published:** 2026-09-18

**Authors:** Mohamad Yousef Alabdallat, Omar Al Ta’ani, Tarek Bakain, Ahmad Al-Dwairy, Mohammad Ghannam, Abdulrahman Atasi, Fares Mashal, Khaled Moghib, Nader Bakheet, Javed G. Jagroo, Rohan Hehr, Ahmed Salem, Derrick Cheung

**Affiliations:** 1 Brookdale University Hospital and Medical Center, Brooklyn, NY, United States; 2 Allegheny General Hospital, Allegheny Health Network, Pittsburgh, PA, United States; 3 Washington University in St. Louis, St. Louis, MO, United States; 4 Georgetown University Hospital, Washington, DC, United States; 5 University of Arkansas for Medical Sciences, Little Rock, AR, United States; 6 Faculty of Medicine, Kasr Al-Ainy, Cairo University, Cairo, Egypt; 7 Maimonides Medical Center, Brooklyn, NY, United States

**Keywords:** acute severe ulcerative colitis, ciclosporin, colectomy, infliximab, JAK inhibitors, network meta-analysis, steroid refractory, tacrolimus

## Abstract

**Background:**

Steroid-refractory acute severe ulcerative colitis is a medical emergency associated with a substantial risk of colectomy. Although infliximab and calcineurin inhibitors are established rescue treatments, comparative evidence regarding alternative drugs, infliximab dosing strategies, and newer small-molecule therapies remains limited. We conducted a systematic review and network meta-analysis to compare the efficacy and safety of available medical rescue strategies.

**Methods:**

PubMed/MEDLINE, Embase, Scopus, Web of Science, and Cochrane CENTRAL were searched from inception through June 30, 2026. Randomized controlled trials and comparative observational studies enrolling adults hospitalized with steroid-refractory acute severe ulcerative colitis were eligible. Separate frequentist random-effects network meta-analyses were conducted for colectomy at approximately 12–14 weeks, colectomy during hospitalization or within 30 days, colectomy at approximately 12 months, and serious adverse events. Effects were expressed as odds ratios (ORs) with 95% confidence intervals (CIs). Risk of bias was assessed using RoB 2 and ROBINS-I.

**Results:**

Of 5, 498 records identified, 18 studies involving 1, 529 participants met the eligibility criteria, including three randomized trials and 15 observational studies. At approximately 12–14 weeks, ciclosporin was associated with higher odds of colectomy than standard-dose infliximab (OR 2.19, 95% CI 1.17–4.12). Neither high-dose infliximab (OR 2.92, 95% CI 0.42–20.26) nor accelerated infliximab (OR 4.57, 95% CI 0.88–23.75) differed significantly from standard-dose infliximab. Ciclosporin was also associated with higher odds of early colectomy (OR 3.05, 95% CI 1.38–6.77) and 12-month colectomy (OR 3.65, 95% CI 1.98–6.72) than standard-dose infliximab. Tacrolimus and accelerated infliximab did not differ significantly from standard-dose infliximab at either time point. Serious adverse events were similar between ciclosporin and infliximab (OR 0.94, 95% CI 0.54–1.63). The networks contained no closed loops, and most observational studies were judged to have a serious risk of bias.

**Conclusions:**

In the combined randomized and observational evidence, standard-dose infliximab was associated with lower colectomy odds than ciclosporin, without a detectable difference in serious adverse events. No consistent advantage was demonstrated for accelerated or high-dose infliximab. Because the apparent efficacy advantage of infliximab was driven predominantly by observational studies, whereas randomized trials showed comparable outcomes, the findings should be interpreted cautiously. Further adequately powered randomized comparisons incorporating contemporary biologic-exposed populations and newer small-molecule therapies are required.

**Systematic review registration:**

https://www.crd.york.ac.uk/prospero/, identifier CRD420261412884.

## 1 Introduction

Acute severe ulcerative colitis (ASUC) is a potentially life-threatening presentation of ulcerative colitis characterized by frequent bloody stools accompanied by systemic toxicity and requiring urgent hospitalization (1, 2). It develops in up to one-quarter of patients with ulcerative colitis during their disease course and remains associated with substantial morbidity, risk of colectomy, and prolonged hospitalization despite advances in inflammatory bowel disease care (2). Intravenous corticosteroids remain the recommended initial treatment; however, approximately 30%–40% of patients fail to achieve an adequate response after 3–5 days and are classified as having steroid-refractory ASUC (2–4). In this setting, timely initiation of medical rescue therapy or surgery is essential because ineffective medical treatment and delayed colectomy may increase the risk of complications.

Infliximab and calcineurin inhibitors, particularly ciclosporin, are the most established medical rescue therapies for steroid-refractory ASUC (3, 4). The CySIF randomized trial found no significant difference in treatment failure between infliximab and ciclosporin at 98 days, while the pragmatic CONSTRUCT trial similarly demonstrated no meaningful difference in colectomy, quality-adjusted survival, or serious adverse events during longer-term follow-up (5, 6). A previous network meta-analysis restricted largely to randomized evidence also concluded that both therapies reduced short-term colectomy compared with placebo, without establishing clear superiority of one active rescue therapy over the other (7). Nevertheless, treatment selection in clinical practice is influenced by previous biologic exposure, disease severity, serum albumin, local expertise, contraindications, the need for drug-level monitoring, and options for subsequent maintenance therapy.

Several developments have further complicated the therapeutic landscape. Accelerated or high-dose infliximab induction has been proposed because severe inflammatory burden, hypoalbuminemia, and intestinal loss of infliximab may increase drug clearance in ASUC. However, observational studies have produced conflicting results, and the PREDICT-UC randomized trial did not demonstrate superiority of an initial 10-mg/kg dose or accelerated induction over standard-dose infliximab for clinical response, remission, or colectomy by 3 months (8). Rapidly acting Janus kinase inhibitors have also emerged as potential rescue options, particularly in biologic-experienced patients. Tofacitinib was associated with a lower adjusted 90-day colectomy risk than matched standard-care controls in a retrospective case-control study, while a recent international comparison of tofacitinib and upadacitinib found similar intermediate-term colectomy-free survival and safety after adjustment for measured confounders (9, 10). These findings remain limited by observational designs, heterogeneous treatment indications, small treatment groups, and differences between first-line and sequential rescue settings.

Previous evidence syntheses have either focused predominantly on infliximab versus ciclosporin, emphasized short-term colectomy, or combined heterogeneous evidence across treatment lines and dosing strategies (7, 11). Moreover, the publication of recent comparative studies involving optimized infliximab regimens, tacrolimus, tofacitinib, and upadacitinib warrants an updated assessment of both early and longer-term outcomes. Therefore, we conducted a systematic review and network meta-analysis of randomized controlled trials and comparative observational studies to evaluate the relative efficacy and safety of available medical rescue strategies for adults with steroid-refractory ASUC. The principal outcomes were early colectomy during hospitalization or within 30 days, short-term colectomy at approximately 12–14 weeks, colectomy at approximately 12 months, and serious adverse events.

## 2 Methods

This study was conducted following the Cochrane Handbook for Systematic Reviews of Interventions (12). The results were reported as specified by the Preferred Reporting Items for Systematic Reviews and Meta-Analyses (PRISMA) statement (13). Additionally, this study has been registered with PROSPERO under the identification number (CRD420261412884).

### 2.1 Information sources and search strategy

The literature search used five electronic databases: Cochrane, PubMed, Web of Science, Embase, and Scopus. The search targeted literature from inception to June 30, 2026. The complete search strategy for each database is provided in the **Supplementary Appendix Table A1**. Two reviewers independently performed the database searches. The reference lists of included studies and relevant review articles were also screened manually to identify additional eligible studies not retrieved through the electronic database search.

The electronic database search was supplemented by manual screening of the reference lists of included studies and relevant reviews. Dedicated grey-literature databases and conference proceedings were not systematically searched because eligibility was restricted to complete peer-reviewed full-text comparative studies. Accordingly, conference abstracts and supplement-only reports without a corresponding full-text peer-reviewed publication were excluded.

### 2.2 Eligibility criteria

Studies were selected according to prespecified eligibility criteria based on the Population, Intervention, Comparator, Outcomes, and Study design framework.

#### 2.2.1 Inclusion criteria

Studies were selected according to the PICOS framework.

- Populations: Studies enrolling adults aged 18 years or older who were hospitalized with acute severe ulcerative colitis and demonstrated an inadequate response to intravenous corticosteroid therapy were eligible. Acute severe ulcerative colitis was required to be defined using the Truelove and Witts criteria, modified Truelove and Witts criteria, Lichtiger score, Oxford criteria, or an explicitly reported equivalent clinical definition. Studies including mixed ulcerative colitis populations were eligible only when data for patients with steroid-refractory acute severe ulcerative colitis could be extracted separately.
- Intervention: Eligible interventions included medical rescue therapies administered for steroid-refractory acute severe ulcerative colitis, including standard-dose infliximab, accelerated infliximab induction, high-dose infliximab, ciclosporin, tacrolimus, tofacitinib, upadacitinib, and other eligible biologic, calcineurin-inhibitor, or small-molecule rescue treatments identified during the search. Standard-dose infliximab was defined as an induction regimen of 5 mg/kg administered at weeks 0, 2, and 6. Accelerated infliximab induction was defined as a shortened induction schedule in which the second and/or third infliximab infusion was administered earlier than the conventional week-2 and/or week-6 schedule. Regimens characterized primarily by administration of a higher infliximab dose, generally 10 mg/kg rather than 5 mg/kg, were classified separately as high-dose infliximab. Because the precise accelerated schedules varied across studies, the study-specific definition was retained when it met the general criterion of a shortened induction interval, and the exact regimen used in each study was reported in **Table 1**.
- Comparator: Eligible comparators included another active rescue therapy, placebo or continued standard medical therapy, matched standard-care controls, or an alternative dose, induction schedule, or timing strategy of the same rescue treatment.
- Outcomes: Studies were required to report colectomy, colectomy-free survival, time to colectomy, or sufficient data from which an eligible comparative treatment effect could be derived.

**Table 1:** Study characteristics and design.

Study	Country/setting	Design and study period	Population and sample size	Intervention	Comparator	Follow-up and relevant outcomes
Lichtiger et al., 1994 (41)	United States; 2 hospitals	Randomized, double-blind, placebo-controlled trial; 1991–1992	Steroid-refractory severe UC; n=20	IV ciclosporin 4 mg/kg/day by continuous infusion for up to 14 days; n=11	Matching placebo plus continued corticosteroid treatment; n=9	14–20-day treatment period; clinical response, colectomy and adverse events
Laharie et al., 2012 (5)	France, Spain, Belgium and Finland; 27 centers	Multicenter, open-label, parallel-group RCT; 2007–2010	Adults with severe UC refractory to ≥5 days of IV corticosteroids; n=115	IV ciclosporin 2 mg/kg/day for 7 days, followed by oral ciclosporin to day 98; n=58	Infliximab 5 mg/kg on days 0, 14 and 42; n=57	98 days; treatment failure, colectomy-free survival, response, remission, mucosal healing and safety
Williams et al., 2016, CONSTRUCT (6)	England, Scotland and Wales; 52 hospitals	Pragmatic, open-label, parallel-group RCT; 2010–2013	Adults with ASUC failing 2–5 days of IV hydrocortisone; n=270	Infliximab 5 mg/kg at weeks 0, 2 and 6; n=135	IV ciclosporin 2 mg/kg/day for up to 7 days, followed by oral ciclosporin 5.5 mg/kg/day for 12 weeks; n=135	1–3 years; quality-adjusted survival, colectomy, mortality, length of stay and adverse events
Croft et al., 2013 (30)	Australia; tertiary referral center	Prospective comparative cohort; 1999–2009	Steroid-refractory ASUC; n=83	IV ciclosporin 2–4 mg/kg/day; n=45	Infliximab 5 mg/kg rescue therapy; n=38	Discharge, 3 and 12 months; colectomy-free survival and serious adverse events
Dean et al., 2012 (31)	New Zealand; single center	Retrospective comparative cohort; 2001–2010	Hospitalized steroid-refractory severe UC; n=38	IV ciclosporin; n=19	Infliximab 5 mg/kg; n=19	12 months; colectomy at 3 and 12 months, length of stay, steroid dependence and adverse events
Kim et al., 2015 (33)	South Korea; single center	Retrospective cohort; 1995–2012	Severe corticosteroid-refractory UC; n=43	IV ciclosporin 2 mg/kg/day followed by oral treatment; n=10	Infliximab 5 mg/kg induction, generally at weeks 0, 2 and 6; n=33	At least 12 months; colectomy-free survival, remission, length of stay, steroid dependence and adverse events
Mocciaro et al., 2012 (35)	Italy; single center	Retrospective historical-cohort study; 1994 onward	Severe steroid-refractory UC; n=65	IV ciclosporin 2 mg/kg/day followed by oral ciclosporin; n=35	Infliximab 5 mg/kg induction and maintenance in responders; n=30	Minimum 12 months; colectomy at 3 and 12 months and long-term follow-up, hospitalization and adverse events
Naves et al., 2014 (37)	Spain; 3 referral centers	Retrospective multicenter cohort; 2005–2011	Steroid-refractory severe UC attacks; n=50	Ciclosporin-based rescue strategy; n=20	Infliximab-based rescue strategy; n=30	Median approximately 3–4 years; colectomy, second-line rescue, relapse and treatment strategy failure
Song et al., 2021 (38)	South Korea; single center	Retrospective cohort; 1995–2015	Steroid-refractory ASUC; n=121	Ciclosporin rescue therapy; n=23	Infliximab rescue therapy; n=98	Median 45 months; treatment failure and colectomy at 3 months and during long-term follow-up
Sror et al., 2026 (39)	Israel; tertiary center	Retrospective comparative cohort; 2017–2024	IV-glucocorticoid-refractory ASUC; n=61	Infliximab rescue therapy; n=37	Tacrolimus rescue therapy with therapeutic drug-level monitoring; n=24	Index hospitalization and 1 year; colectomy-free response, colectomy, treatment failure, length of stay and adverse events
Gibson et al., 2015 (32)	Ireland; single center	Retrospective before-and-after cohort; 2005–2013	Steroid-refractory ASUC receiving infliximab; n=50	Accelerated infliximab: three 5-mg/kg doses within a median of 24 days; n=15	Standard infliximab 5 mg/kg at weeks 0, 2 and 6; n=35	Induction period and approximately 2 years; early and long-term colectomy
Choy et al., 2018 (29)	Australia; 7 centers	Retrospective multicenter cohort; 2014–2015	Hospitalized ASUC receiving infliximab rescue; n=54	Accelerated infliximab, with second and third 5-mg/kg doses administered earlier according to clinical need	Standard 5-mg/kg infliximab at weeks 0, 2 and 6	12 months; colectomy and predictors of infliximab failure
Chao et al., 2019 (28)	Canada; 2 hospitals	Retrospective cohort; 2010–2016	Hospitalized steroid-refractory ASUC; n=72	Initial high-dose infliximab 10 mg/kg; n=35	Standard-dose infliximab 5 mg/kg; n=37	30 days, 3 months and 2 years; colectomy-free survival and predictors of colectomy
Syal et al., 2021 (40)	United States; single center	Retrospective cohort; 2012–2017	ASUC failing 3–5 days of IV corticosteroids; n=63	Infliximab 10 mg/kg; n=34	Infliximab 5 mg/kg; n=29	90 days and 1 year; colectomy and predictors of infliximab rescue failure
Liu et al., 2024 (34)	China; 7 tertiary centers	Retrospective multicenter propensity-adjusted cohort; 2012–2022	Steroid-refractory ASUC receiving ≥2 infliximab doses; n=76	Accelerated/intensified induction; n=47	Standard infliximab induction; n=29	Day 14, 90 days and long-term follow-up; remission, colectomy and time-to-colectomy
Mohamedrashed et al., 2026 (36)	Australia; single tertiary center	Retrospective cohort; 2016–2024	ASUC with inadequate response to IV corticosteroids; n=84	Early infliximab initiation ≤72 hours after admission; n=31	Later infliximab initiation >72 hours after admission; n=53	12 months; length of stay, index and 12-month colectomy, readmission and infection
Berinstein et al., 2021 (9)	United States; single academic center	Retrospective matched case-control study	Biologic-experienced patients hospitalized with ASUC; n=153	Tofacitinib, usually 10 mg twice or three times daily, plus IV corticosteroids; n=40	Matched hospitalized controls receiving IV corticosteroids and standard rescue care; n=113	90 days; colectomy, steroid dependence and complications
Honap et al., 2026 (10)	International; 23 centers	Retrospective multicenter propensity-adjusted cohort; 2019–2024	Hospitalized adults treated with JAK inhibitors for ASUC; n=111; strict salvage subgroup n=66	Tofacitinib; total n=60, salvage subgroup n=35	Upadacitinib; total n=51, salvage subgroup n=31	Days 3–7, week 14 and week 26; clinical response, remission, colectomy-free survival and safety

The primary outcome was short-term colectomy at approximately 12–14 weeks after initiation of rescue therapy.

The principal secondary outcomes were:

- Early colectomy, defined as colectomy during the index hospitalization or within 30 days of rescue-therapy initiation.
- Colectomy at approximately 12 months after rescue-therapy initiation.
- Serious adverse events during the reported treatment-comparable follow-up period.

Additional outcomes of interest included clinical response, clinical remission, treatment failure, need for additional rescue therapy, length of hospitalization, readmission, infection, mortality, and other reported safety outcomes. When studies reported time-to-event estimates or colectomy-free survival without sufficiently reliable binary event data for the prespecified time window, these estimates were summarized separately and were not converted into artificial event counts solely for inclusion in the network meta-analysis.

- Study types: Randomized controlled trials and comparative nonrandomized studies, including prospective or retrospective cohort studies and case-control studies, were eligible. Only complete peer-reviewed full-text articles were included.

#### 2.2.2 Exclusion criteria

Studies were excluded if they met any of the following criteria: nonhuman or *in vitro* studies; pediatric-only studies; studies not involving hospitalized patients with acute severe ulcerative colitis; studies in which corticosteroid refractoriness was not established or relevant subgroup data were unavailable; studies evaluating outpatient moderate-to-severe ulcerative colitis without an acute severe hospitalized population; noncomparative studies, single-arm case series, or case reports; narrative reviews, systematic reviews, editorials, commentaries, protocols, or letters without original data; conference abstracts or supplement-only reports without a corresponding complete peer-reviewed publication; studies evaluating surgical, nutritional, or other noneligible interventions without an eligible medical rescue-therapy comparison; studies without an eligible comparator group; studies reporting no extractable colectomy or colectomy-free-survival outcome; and studies with insufficient information for reliable data extraction after full-text assessment. When multiple publications reported data from the same or substantially overlapping population, the most complete or most recent report was retained for each outcome and follow-up period. Companion publications were used only to supplement missing information and were not counted as independent studies.

### 2.3 Study selection and data management

All retrieved records were exported to reference-management software, and duplicate records were removed before screening. The screening process was conducted in two phases. First, two reviewers independently screened titles and abstracts to identify potentially eligible studies. Second, the full texts of potentially relevant articles were reviewed independently by the same two reviewers to confirm eligibility. Rayyan software was used to facilitate the screening process. Disagreements were resolved through discussion, and unresolved conflicts were adjudicated by a third reviewer (14).

### 2.4 Data extraction

Two reviewers independently extracted data from each eligible study using a standardized and piloted data-extraction form. Extracted information included the first author, publication year, country, number of participating centers, study design, recruitment period, eligibility criteria, definition of acute severe ulcerative colitis, definition and duration of corticosteroid failure, sample size, treatment groups, rescue-line setting, intervention dose, administration route, induction schedule, treatment timing, comparator strategy, and duration of follow-up. Participant-level characteristics extracted included age, sex, disease duration, disease extent, previous exposure to biologic or small-molecule therapy, concomitant immunomodulator use, stool frequency, clinical disease-activity scores, endoscopic severity, C-reactive protein, serum albumin, hemoglobin, and other reported markers of baseline disease severity. For efficacy outcomes, the reviewers extracted the number of colectomy events, total number of participants in each group, reported colectomy-free-survival probabilities, time to colectomy, hazard ratios, risk ratios, odds ratios, and corresponding 95% confidence intervals. Outcomes were extracted at all reported time points, including during the index hospitalization, approximately 4 weeks, 12 weeks, 6 months, 12 months, and the longest available follow-up. When time-to-event estimates were unavailable but Kaplan–Meier curves were presented, survival information was extracted for potential reconstruction using established methods. For nonrandomized studies, adjusted effect estimates were preferred over unadjusted estimates when both were available. The variables included in each adjusted model were also recorded. Safety data included any adverse event, serious adverse event, infection, serious infection, treatment discontinuation, postoperative complications, and mortality. Where necessary, information reported in supplementary materials or companion publications was used to complete missing study-level data. Discrepancies between reviewers were resolved by consensus, with referral to a third reviewer when required. No numerical data were imputed when the required information could not be reliably derived from the publication.

### 2.5 Risk of bias assessment

Two reviewers independently assessed the risk of bias of included studies. Randomized controlled trials were evaluated using the revised Cochrane Risk of Bias tool for randomized trials (RoB 2). The assessed domains included bias arising from the randomization process, bias due to deviations from intended interventions, bias due to missing outcome data, bias in outcome measurement, and bias in the selection of the reported result. Each domain was judged as low risk, some concerns, or high risk of bias, and an overall judgment was assigned for each randomized trial (15).

Non-randomized studies were assessed using the Risk Of Bias In Non-randomized Studies of Interventions tool (ROBINS-I). The assessed domains included bias due to confounding, bias in the selection of participants, bias in the classification of interventions, bias due to deviations from intended interventions, bias due to missing data, bias in the measurement of outcomes, and bias in the selection of the reported result. Overall judgments were categorized as low, moderate, serious, critical risk of bias, or no information (16).

Disagreements between reviewers were resolved by discussion or by consultation with a third reviewer. Risk-of-bias figures were generated using the robvis visualization tool (17).

### 2.6 Publication bias

As indicated by Egger et al., the assessment of potential publication bias within this review utilizing Egger’s test for funnel plot asymmetry is not feasible when the number of included studies is fewer than ten (18).

### 2.7 Choice of the meta-analysis model

Because clinical and methodological heterogeneity was anticipated across studies, all pairwise and network meta-analyses were conducted using random-effects models. Heterogeneity was assumed to be common across treatment comparisons within each network, and the between-study variance τ2\tau^2τ2 was estimated using restricted maximum-likelihood estimation rather than the DerSimonian–Laird method. Separate treatment networks were constructed for the four prespecified outcomes: early colectomy during the index hospitalization or within 30 days, short-term colectomy at approximately 12–14 weeks, colectomy at approximately 12 months, and serious adverse events. Standard-dose infliximab was selected as the reference treatment because it was the most frequently studied intervention and provided the principal connection between treatment nodes.

Treatments were represented as distinct nodes according to their clinical strategy. Standard-dose, accelerated, and high-dose infliximab were therefore analyzed separately. Ciclosporin, tacrolimus, tofacitinib, and upadacitinib were also treated as distinct interventions. Comparisons that were clinically or statistically disconnected from the main network, including infliximab-timing strategies and some Janus kinase inhibitor comparisons, were analyzed separately and were not incorporated into an invalid global treatment ranking.The transitivity assumption was evaluated by comparing the distribution of potential effect modifiers across treatment comparisons, including baseline disease severity, serum C-reactive protein, serum albumin, previous biologic or small-molecule exposure, duration of intravenous corticosteroid therapy, rescue-line setting, treatment era, and follow-up duration. Because the resulting networks did not contain closed loops, disagreement between direct and indirect evidence could not be formally assessed using node-splitting or loop-specific inconsistency methods (19).

Because both randomized and nonrandomized comparative studies contributed to the evidence network, the transitivity assumption was assessed with particular attention to study design and potential confounding. The distribution of clinically relevant effect modifiers was compared across treatment contrasts, including baseline disease severity, C-reactive protein, serum albumin, previous biologic or small-molecule exposure, duration of intravenous corticosteroid treatment, rescue-line setting, treatment era, and follow-up duration. Studies were combined only when the population, clinical setting, intervention definition, comparator, and outcome window were considered sufficiently comparable to support a common clinical question. Nevertheless, observational studies remained susceptible to residual confounding by indication and treatment-era effects, and this limitation was considered when interpreting the network estimates.

Prespecified subgroup or sensitivity analyses according to study design were considered; however, these analyses could not be performed reliably because the individual treatment contrasts were informed by very few studies, several treatment nodes were represented by a single study, and randomized evidence was available for only a limited subset of comparisons. Formal meta-regression according to study design was therefore not undertaken because the available number of studies was insufficient to provide stable or meaningful regression estimates.

### 2.8 Calculation of missing data

For dichotomous outcomes, the numbers of events and participants in each treatment group were extracted directly from the published reports. When outcomes were reported at multiple time points, the assessment closest to the prespecified outcome window was selected. Available-case denominators were used when the number of participants assessed at follow-up differed from the original treatment-group size. When adjusted time-to-event estimates were reported, hazard ratios and corresponding 95% confidence intervals were extracted and summarized separately. Kaplan–Meier probabilities were not converted into artificial event counts when the number of events could not be reliably reconstructed. Studies reporting zero events in both treatment groups were retained in the qualitative synthesis but did not contribute to conventional odds-ratio estimation. No participant-level or outcome data were imputed when they could not be reliably obtained or derived from the publication. In instances where data were reported as the median and interquartile ranges (IQR), conversions were performed to obtain mean (M) and standard deviation (SD) utilizing the equations established by Wan et al. (20). Furthermore, when the standard deviation was not supplied, it was derived from the standard error using the formula for a single sample: SD = SE*√n, where (n) denotes the sample size (21). If the mean change (MC) between baseline and endpoint data was unavailable, it was calculated from the pre-treatment and post-treatment means using the equation MC = Mpost-treatment - Mpre-treatment. Additionally, in cases where the standard deviation of the mean change was not provided, it was computed from the standard deviations of the pre-treatment and post-treatment samples using the formula SD = √(SD²pre-treatment + SD²post-treatment) (22). Effect sizes (d) and effect size correlations (r) that were missing were calculated through the following formulas: d = (Mtreatment - Mcontrol)/SDpooled, with SDpooled calculated as √[(SD²treatment + SD²control)/2] (22), and r = d/√(d² + 4) (23).

### 2.9 Statistical analysis

Statistical analyses were performed using R software, version 4.4.1. Dichotomous outcomes were summarized as odds ratios (ORs) with 95% confidence intervals (CIs). Colectomy was coded as the event; therefore, an OR below 1 favored the treatment listed first. A two-sided P<0.05P<0.05P<0.05 was considered statistically significant. Random-effects models were used because clinical and methodological heterogeneity was expected across studies. The between-study variance was estimated using the restricted maximum-likelihood method. Standard-dose infliximab was selected as the reference treatment because it was the most frequently studied intervention and connected most treatment nodes. Separate network meta-analyses were performed for each connected outcome network:

1. Short-term colectomy at approximately 12–14 weeks.
2. Early colectomy during hospitalization or within 30 days.
3. Colectomy at approximately 12 months.
4. Serious adverse events.

Comparisons that were disconnected from the main networks, including early versus later infliximab administration and some JAK-inhibitor comparisons, were analyzed separately using direct pairwise estimates and were not included in the overall network rankings. Results were presented using network plots, forest plots, and league tables. In the network plots, node size was proportional to the number of participants receiving each treatment, and line thickness was proportional to the number of studies directly comparing the connected treatments. Treatment rankings were summarized using P-scores, with higher values indicating a greater probability of being among the more favorable treatments. Rankings were interpreted cautiously when estimates were imprecise or based on a single study. Statistical heterogeneity was assessed using Cochran’s Q test, the I2I^2I2 statistic, and the between-study variance τ2\tau^2τ2. A P<0.10P<0.10P<0.10 for Cochran’s Q test was considered suggestive of heterogeneity. I2I^2I2 values of 0%–40%, 30%–60%, 50%–90%, and 75%–100% were interpreted as low, moderate, substantial, and considerable heterogeneity, respectively. Formal assessment of inconsistency between direct and indirect evidence was planned. However, the networks did not contain closed loops; therefore, node-splitting and loop-specific inconsistency analyses could not be performed. Where sufficient data were available, sensitivity analyses were planned by excluding studies at serious risk of bias, studies with substantial loss to follow-up, historical cohorts, and studies requiring continuity corrections for zero-event treatment groups. Randomized and observational evidence was also examined separately when feasible (19, 24–27).

For dichotomous comparisons in which one treatment arm contained zero events, a fixed continuity correction of 0.5 was applied by adding 0.5 to each cell of the corresponding 2 × 2 contingency table before calculation of the log odds ratio and its standard error. Studies reporting zero events in both treatment arms were not estimable using conventional odds-ratio methods and therefore did not contribute to the corresponding pooled effect estimate. This correction was required for the early-colectomy comparison of accelerated versus standard-dose infliximab reported by Liu et al., in which 30-day colectomy occurred in 2 of 47 patients receiving accelerated induction and 0 of 29 patients receiving standard-dose induction.

## 3 Results

### 3.1 Search and screening

The database search identified 5, 498 records: 1, 116 from PubMed/MEDLINE, 1, 294 from Scopus, 1, 581 from Web of Science, 182 from the Cochrane Central Register of Controlled Trials, and 1, 325 from Embase. After removal of 3, 066 duplicate records, 2, 432 unique citations remained for title and abstract screening. Of these, 2, 300 records were excluded because they were clearly unrelated to steroid-refractory acute severe ulcerative colitis, did not evaluate an eligible medical rescue intervention, or did not report relevant comparative clinical outcomes.

A total of 132 reports were retrieved and assessed in full text. No potentially eligible report was unavailable for full-text assessment. Following a detailed eligibility evaluation, 114 reports were excluded because they did not meet the prespecified inclusion criteria. The most frequent reasons were inclusion of patients without confirmed acute severe or steroid-refractory ulcerative colitis, absence of a comparative treatment group, evaluation of non-eligible interventions, lack of colectomy or colectomy-free survival data, case-series design, duplicate or overlapping populations, and publication as a conference abstract without a complete peer-reviewed article. Ultimately, 18 full-text studies (5, 6, 9, 10, 28–41) were included in the systematic review and quantitative synthesis. The study-selection process is presented in **Figure 1**.

**Figure 1:**
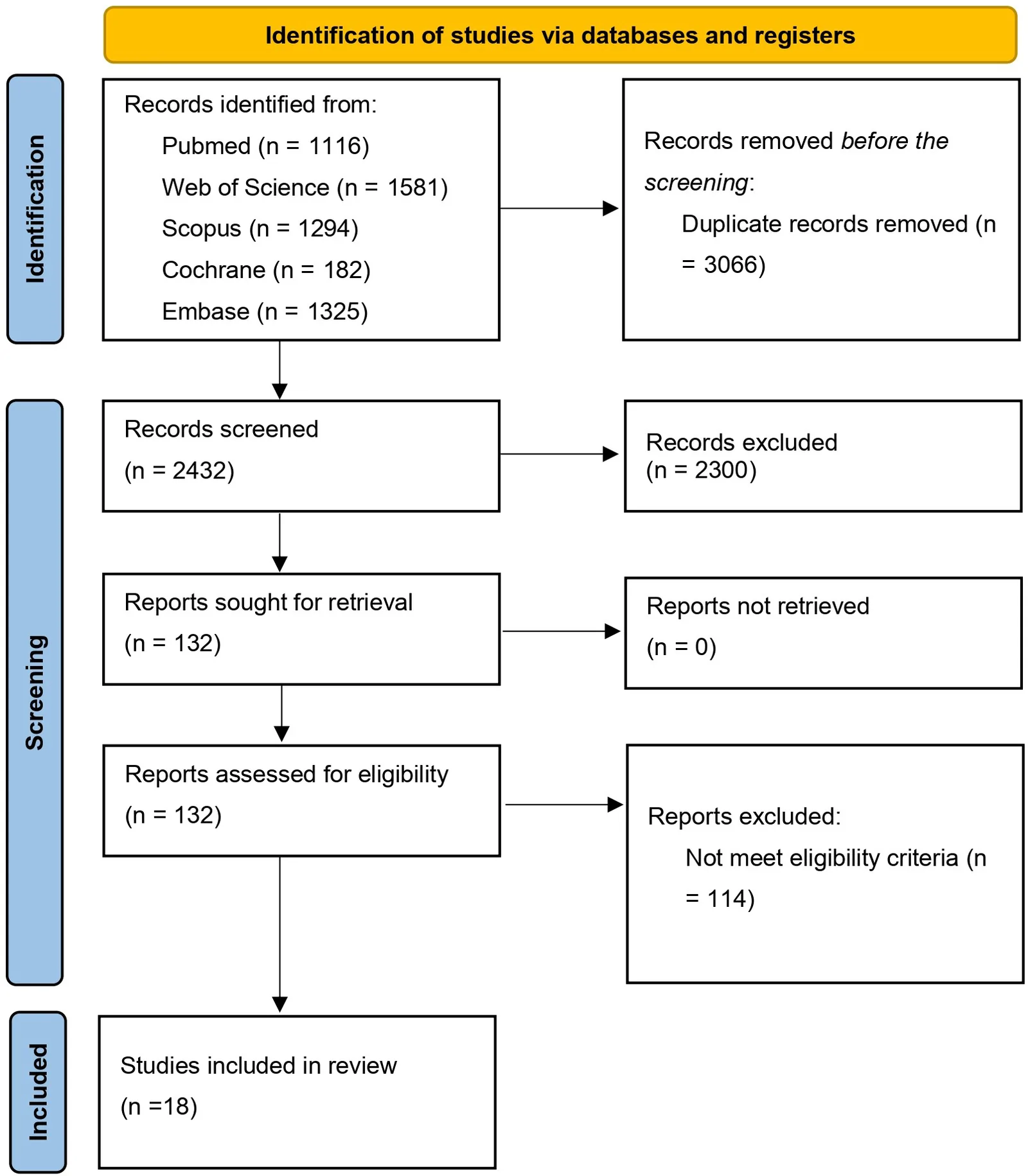
PRISMA flow chart for study screening and inclusion.

### 3.2 Study characteristics

The 18 included studies comprised three randomized controlled trials and 15 comparative observational studies, with reported study-level sample sizes totaling 1, 529 participants. Individual study sizes ranged from 20 participants in the early placebo-controlled ciclosporin trial to 270 participants in the CONSTRUCT randomized trial. The studies were conducted across North America, Europe, Australia, New Zealand, China, South Korea, Israel, and international multicenter settings, providing evidence from geographically and clinically diverse populations.

The three randomized trials evaluated ciclosporin against placebo or infliximab. Lichtiger et al. compared intravenous ciclosporin with placebo in patients with severe ulcerative colitis refractory to intravenous corticosteroids. Laharie et al. directly compared ciclosporin with standard-dose infliximab over 98 days, whereas the pragmatic CONSTRUCT trial compared the same two rescue strategies with longer-term clinical and surgical follow-up.

Six observational studies compared ciclosporin-based and infliximab-based rescue strategies. These cohorts generally included hospitalized adults with severe ulcerative colitis that had failed intravenous corticosteroids and evaluated colectomy during hospitalization, at approximately 3 months, at 12 months, or during longer follow-up periods. Sample sizes ranged from 38 to 121 participants. Although several cohorts reported similar short-term effectiveness between ciclosporin and infliximab, some historical comparisons favored infliximab for colectomy avoidance, particularly at longer follow-up. Differences in treatment era, maintenance therapy, baseline disease severity, and concomitant immunomodulator use were common across these studies.

One recent cohort expanded the conventional rescue-treatment network by comparing infliximab with tacrolimus in 61 adults with intravenous glucocorticoid-refractory ASUC. Despite substantially greater previous advanced-therapy exposure in the tacrolimus group, colectomy-free response at discharge and colectomy during the subsequent year were similar between treatments.

Six studies evaluated alternative infliximab administration strategies rather than different drug classes. These included accelerated versus standard induction, an initial dose of 10 mg/kg versus 5 mg/kg, and infliximab initiated within 72 hours versus later administration. The definitions of accelerated treatment varied across studies and included shortened intervals between infusions, higher initial doses, or both. Follow-up most commonly included colectomy during induction, at 90 days, and at 1 year. Gibson et al. reported fewer early colectomies with accelerated induction, whereas Choy, Chao, Syal, and Liu did not demonstrate a consistent benefit after accounting for baseline disease severity and confounding by indication. Mohamedrashed et al. found that earlier infliximab initiation was associated with a modestly shorter hospital stay but not with lower colectomy or infection rates.

Two studies evaluated Janus kinase inhibitors. Berinstein et al. compared tofacitinib with matched hospitalized controls among biologic-experienced patients and reported a lower adjusted 90-day risk of colectomy with tofacitinib, particularly with the 10-mg three-times-daily regimen. Honap et al. directly compared tofacitinib with upadacitinib in an international cohort. In the strict salvage subgroup, the two agents produced similar colectomy-free survival and safety outcomes at approximately 14 and 26 weeks.

Across the evidence base, colectomy or colectomy-free survival was the principal efficacy outcome. However, outcome definitions and assessment times varied, including colectomy during the index admission, induction period, 30 days, 90 days, 12 months, and longer-term follow-up. Secondary outcomes included clinical response, clinical remission, treatment failure, length of hospitalization, readmission, adverse events, serious adverse events, infections, and mortality.

Because the included studies addressed clinically distinct questions, they were categorized into three analytical groups: comparisons between different rescue agents, comparisons between infliximab dosing or timing strategies, and comparisons involving JAK inhibitors. This separation was necessary to preserve clinical comparability and the transitivity assumption of the network meta-analysis. (**Tables 1**, **2**).

**Table 2:** Baseline demographics and risk scores by treatment arm.

Study	Age, years	Male sex	Disease duration	Extensive colitis	Previous/concomitant therapy	Baseline disease activity and laboratory characteristics
Lichtiger 1994 (41)	34 vs 43, mean	4/11 vs 5/9	6 vs 2 years, mean	8/11 vs 8/9 universal colitis	Concomitant corticosteroids and topical therapy in both groups	Clinical-activity index 13 vs 14; prior parenteral corticosteroids 16 vs 17 days
Laharie 2012 (5)	39 vs 36	52% vs 53%	2.4 vs 1.0 years	59% vs 54%	Azathioprine-naïve: 93% vs 93%	CRP 30 vs 46 mg/L; albumin 28 vs 27 g/L; Hb 105 vs 115 g/L; Mayo endoscopic score 3: 95% vs 96%
Williams 2016 (6)	35 vs 36	66% vs 60%	NR	E3: 43% vs 49%	Immunomodulator at baseline: 12% vs 19%	Severe Truelove–Witts disease: 73% vs 73%; CRP 62 vs 54 mg/L; albumin 33 vs 33 g/L; Hb 12.7 vs 12.6 g/dL
Croft 2013 (13)	32 vs 28	60% vs 39%	3.57 vs 0.34 years	74% vs 71%	Immunosuppressive therapy at admission: 26% vs 13%	CRP 53 vs 72 mg/L; all participants met Truelove and Witts criteria
Dean 2012 (31)	31 vs 25	63% vs 58%	36 vs 12 months	9/19 vs 10/19	Immunomodulator at treatment: 1/19 vs 5/19	CRP 56 vs 32 mg/L; duration of IV steroids was broadly comparable
Kim 2015 (33)	56 vs 44	30.0% vs 75.8%	121.4 vs 76.3 months	80.0% vs 36.4%	Azathioprine at treatment: 40.0% vs 84.8%	CRP 10.9 vs 30.0 mg/L
Mocciaro 2012 (35)	34.9 ± 13.7 vs 37.0 ± 16.6	42.8% vs 50.0%	36 vs 48 months	83% vs 66.7%	Previous azathioprine/6-MP: 42% vs 30%	Hb 9.2 ± 0.7 vs 9.4 ± 0.5 g/dL; all had Lichtiger score >10
Naves 2014 (37)	42 vs 38	65% vs 57%	42 vs 37 months	70% vs 70%	Prior thiopurine: 15% vs 33%	Severe activity immediately before rescue: 50% vs 20%; IV steroid duration 7 vs 9 days
Song 2021 (38)	34.4 vs 34.5 at diagnosis	60.9% vs 54.1%	27.1 vs 48.8 months	52.2% vs 63.3%	Previous azathioprine: 39.1% vs 40.8%	Mayo score 10 vs 10; CRP 2.1 vs 1.4 mg/dL; IV steroid duration 12 vs 13 days
Sror 2026 (39)	27 vs 32	32.4% vs 41.7%	1 vs 4 years	54.1% vs 70.8%	Prior advanced therapy: 13.5% vs 62.5%; prior anti-TNF: 2.7% vs 58.3%	CRP 70.0 vs 53.7 mg/L; albumin 32.78 ± 5.30 vs 31.80 ± 6.30 g/L; UCEIS 5 vs 6
Gibson 2015 (32)	34 vs 38	43% vs 53%	1.02 vs 0.45 years	NR	Thiopurine exposure varied	CRP 67.1 vs 32 mg/L; albumin 22 vs 23 g/L; Hb 11.2 vs 11.0 g/dL; IV steroids 7 vs 7 days
Choy 2018 (8)	Overall median approximately 33	Overall 64.8%	Approximately 2.5 years overall	NR	Immunomodulator use was common	Overall cohort: 54 patients; accelerated patients had more severe biochemical disease; Mayo endoscopic score 3 was associated with colectomy
Chao 2019 (28)	Overall 38.5	Overall 62.5%	Overall 3 years	91.7% pancolitis	Immunomodulator before admission: 38.9%; prior biologic: 2.8%	Mayo score 12; endoscopic Mayo 3; CRP 46.5 mg/L; albumin 30 g/L; Hb 106 g/L
Syal 2021 (40)	Overall approximately 37	Overall 44%	NR	84% pancolitis	Previous non-infliximab anti-TNF: 21%; concomitant immunomodulator: 43%	Patients receiving 10 mg/kg generally had lower albumin and greater inflammatory burden
Liu 2024 (34)	43 ± 14.9 vs 40 ± 15.6	75.9% vs 46.8%	2.1 vs 1.6 years	96.6% vs 91.3%	Previous biologic/small molecule: 13.8% vs 8.5%	CRP at first infliximab 12.2 vs 25.9 mg/L; clinical Mayo subscore 8 vs 9; UCEIS 6 vs 7
Mohamedrashed 2026 (36)	34 vs 35	61.3% vs 64.1%	NR	NR	Prior advanced therapy: 16.1% vs 13.2%	CRP 36 vs 40 mg/L; albumin 36 vs 32.5 g/L; Hb 130 vs 133 g/L; UCEIS 7 vs 7; initial 10-mg/kg dose: 83.8% vs 45.3%
Berinstein 2021 (9)	34.4 vs 38.4	40.0% vs 48.7%	10.35 vs 8.41 years	77.5% vs 70.5%	Prior biologic: 100% vs 38.9%; previous infliximab failure: 85.0% vs 27.4%	CRP 6.29 vs 7.71 mg/dL; albumin 3.40 vs 3.28 g/dL; Hb 11.71 vs 12.07 g/dL; Mayo endoscopic score 3: 71.8% vs 55.4%
Honap 2026 (10)	35 vs 34	66% vs 49%	5 vs 4 years	Extent greater than proctitis: 97% vs 100%	Prior anti-TNF: 90% vs 76%; previous infliximab rescue: 40% vs 24%	CRP 52 vs 35 mg/L; albumin 33 vs 32 g/L; Hb 115 vs 113 g/L; Mayo endoscopic score 3: 73% vs 69%

### 3.3 Risk of bias assessment

Risk of bias was assessed separately according to study design. The three randomized controlled trials were evaluated using the Cochrane RoB 2 tool, whereas the 15 nonrandomized comparative studies were assessed using ROBINS-I. Assessments were performed for the outcome of colectomy or colectomy-free survival.

All three randomized trials were judged to have some concerns, and none was classified as having a high overall risk of bias. The Lichtiger trial was double-blinded and placebo-controlled, resulting in a low risk of bias from deviations from the intended interventions and outcome measurement. Nevertheless, the published report provided limited information regarding sequence generation, allocation concealment, and prespecification of the colectomy outcome, leading to some concerns regarding the randomization process and selective reporting.

The Laharie and CONSTRUCT trials used adequate centralized or protected randomization methods and had little evidence of important missing outcome data. However, both were open-label. Knowledge of allocated treatment could have influenced decisions regarding additional rescue treatment, treatment discontinuation, or the timing of colectomy, even though colectomy itself was an objectively verifiable event. Consequently, both trials were judged to have some concerns arising primarily from deviations from intended interventions and potential influence on clinical outcome decisions.

Among the 15 observational studies, one study was judged to have a moderate overall risk of bias and 14 were judged to have a serious risk of bias. No study was judged to have a critical risk of bias. The principal limitation was confounding by indication. Patients with more severe inflammatory activity, lower albumin, higher C-reactive protein, previous biologic failure, or a more treatment-refractory disease course were more likely to receive accelerated or high-dose infliximab, tacrolimus, tofacitinib, or upadacitinib. This imbalance could bias unadjusted comparisons against the more intensive intervention.

Historical ciclosporin-versus-infliximab cohorts were additionally vulnerable to differences in treatment era, supportive care, maintenance strategies, surgical thresholds, and availability of biologic therapy. Several studies compared patients treated during largely nonoverlapping calendar periods, increasing the possibility that observed differences reflected changes in overall management rather than the rescue therapy alone.

Liu et al. was judged to have a moderate overall risk of bias, because the analysis used propensity-score adjustment and adjustment for treating institution to reduce differences between accelerated and standard infliximab groups. Nevertheless, the retrospective design and possibility of residual unmeasured confounding prevented classification as low risk.

The remaining observational studies were judged to have a serious overall risk of bias despite the use of multivariable adjustment, matching, or inverse-probability weighting in some reports. In Berinstein et al., all tofacitinib-treated patients had previous biologic exposure, whereas substantially fewer controls were biologic experienced. In Honap et al., treatment selection between tofacitinib and upadacitinib remained clinically determined despite propensity-adjusted analyses. Similarly, the tacrolimus group in Sror et al. had substantially greater previous anti-TNF and JAK-inhibitor exposure than the infliximab group.

Risk related to outcome measurement was generally low because colectomy was objectively recorded. However, the decision to proceed to surgery remained partly dependent on clinician judgment, local expertise, patient preference, and response to additional medical treatment. Missing outcome data were usually limited, although several studies had incomplete long-term follow-up or reduced denominators at later time points. Selective-reporting concerns were generally moderate because most observational studies did not provide a publicly available prespecified analysis plan.

Overall, the randomized evidence provided greater internal validity but remained limited by open-label treatment allocation and small numbers of trials. The observational evidence substantially increased the size and connectivity of the treatment network but was limited principally by residual confounding, baseline treatment-group imbalance, and heterogeneity in rescue-treatment selection. These limitations were considered when interpreting treatment rankings and assessing the certainty of the network estimates. (**Figure 2**).

**Figure 2:**
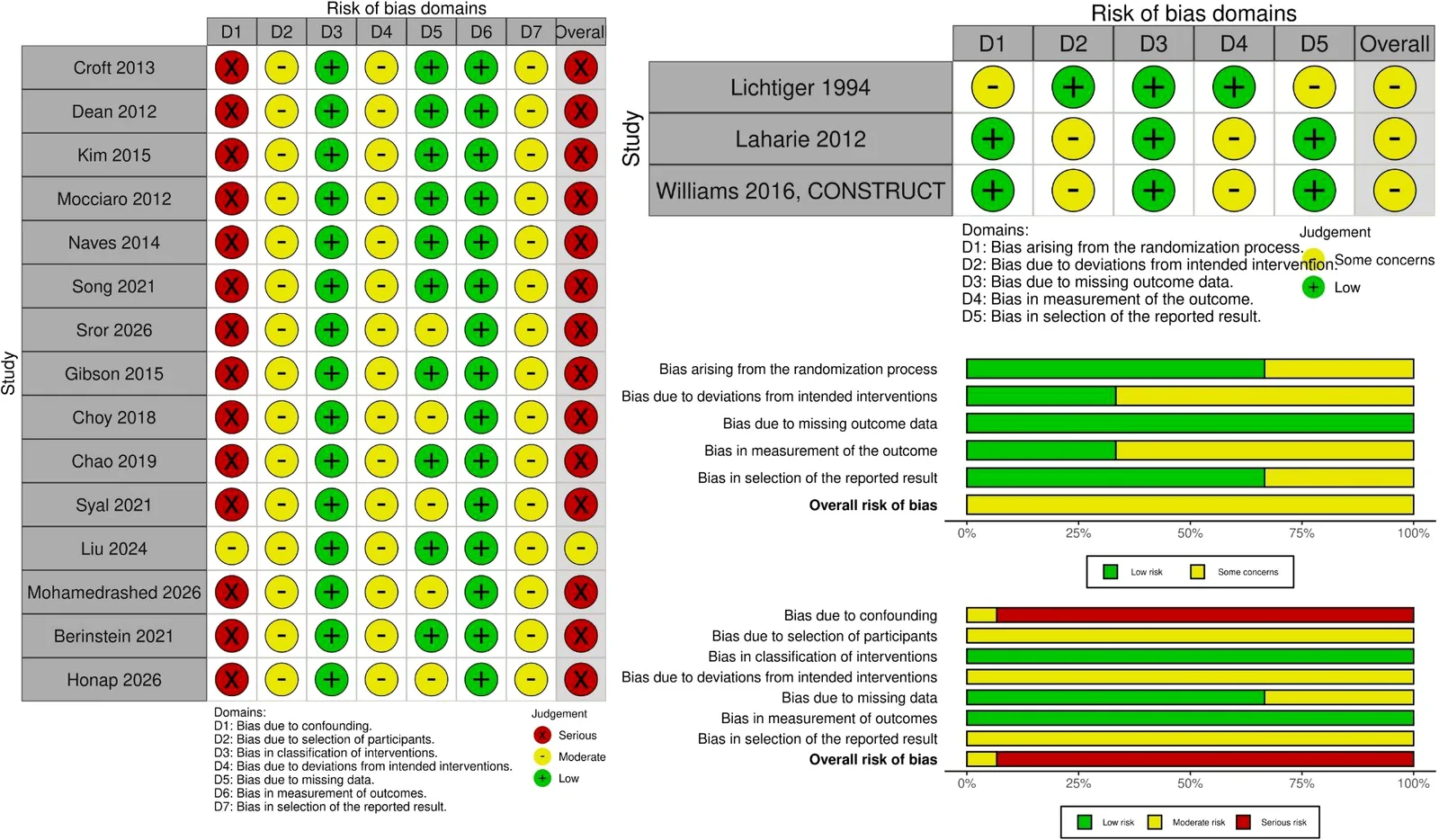
‘Risk of bias graph’: review authors’ judgments about each risk of bias item presented as percentages across all included studies, and ‘Risk of bias summary’: review authors’ judgments about each risk of bias item for each included study.

### 3.4 Outcomes

#### 3.4.1 Short-term colectomy

Nine studies involving 657 participants contributed to the connected network for colectomy at approximately 12–14 weeks. The network included conventional or standard-dose infliximab, ciclosporin, accelerated infliximab, and high-dose infliximab. Moderate between-study heterogeneity was detected (τ²=0.216; I²=30.5%; Cochran Q = 8.63, p=0.196).

Compared with conventional infliximab, ciclosporin was associated with significantly higher odds of short-term colectomy (OR 2.19, 95% CI 1.17–4.12; p=0.015). Neither accelerated infliximab (OR 4.57, 95% CI 0.88–23.75) nor high-dose infliximab (OR 2.92, 95% CI 0.42–20.26) was significantly different from conventional infliximab. The wide confidence intervals surrounding the intensified-regimen estimates reflected the small number of studies, low event counts, and imprecision of these comparisons.

Conventional infliximab had the highest P-score for preventing short-term colectomy (0.94), followed by ciclosporin (0.47), high-dose infliximab (0.39), and accelerated infliximab (0.20). These rankings should be interpreted cautiously because the network contained no closed loops and the intensified-infliximab nodes were each informed by only one or two observational comparisons.

Importantly, the apparent benefit of infliximab over ciclosporin was driven predominantly by nonrandomized evidence. In the randomized Laharie trial, the direct comparison was neutral (OR for ciclosporin vs infliximab 0.78, 95% CI 0.31–1.99), whereas the pooled observational comparisons favored infliximab (OR 2.88, 95% CI 1.64–5.06). The randomized trial reported similar colectomy-free survival between treatments, while several observational cohorts reported lower short-term colectomy rates with infliximab. This divergence suggests possible confounding by treatment era, baseline disease severity, concomitant maintenance therapy, and selection of rescue treatment.

##### 3.4.1.1 Separate JAK-inhibitor evidence

The JAK-inhibitor studies could not be incorporated into the main network because they lacked a common, clearly defined comparator connecting them to infliximab or ciclosporin. Berinstein et al. reported that tofacitinib was associated with a lower adjusted 90-day colectomy risk than matched standard-care controls (adjusted HR 0.28, 95% CI 0.10–0.81). However, the crude binary comparison was less precise, and the tofacitinib group consisted entirely of biologic-experienced patients receiving concomitant intravenous corticosteroids.

Honap et al. found nearly identical colectomy-free survival with tofacitinib and upadacitinib at day 98—79% and 80%, respectively—and no difference between the survival curves (p=0.99). Because the study reported Kaplan–Meier probabilities rather than a comparative hazard ratio, these estimates were not converted into artificial binary event counts (**Figures 3**, **4**).

**Figure 3:**
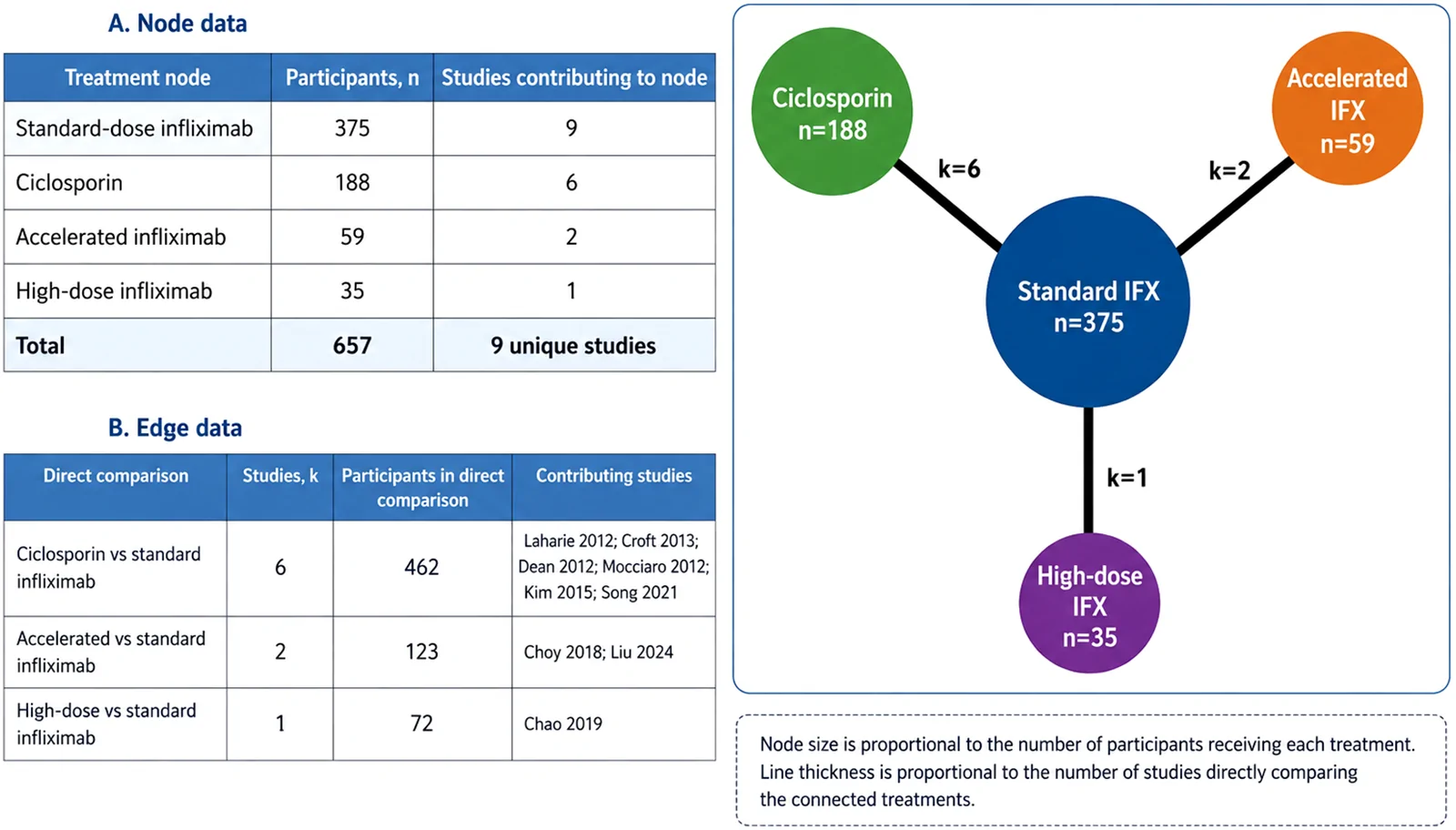
Network geometry for short-term colectomy after rescue therapy. Node size is proportional to the number of participants receiving each treatment, and line thickness is proportional to the number of studies directly comparing the connected interventions. The network included nine studies and 657 participants. No closed evidence loop was present.

**Figure 4:**
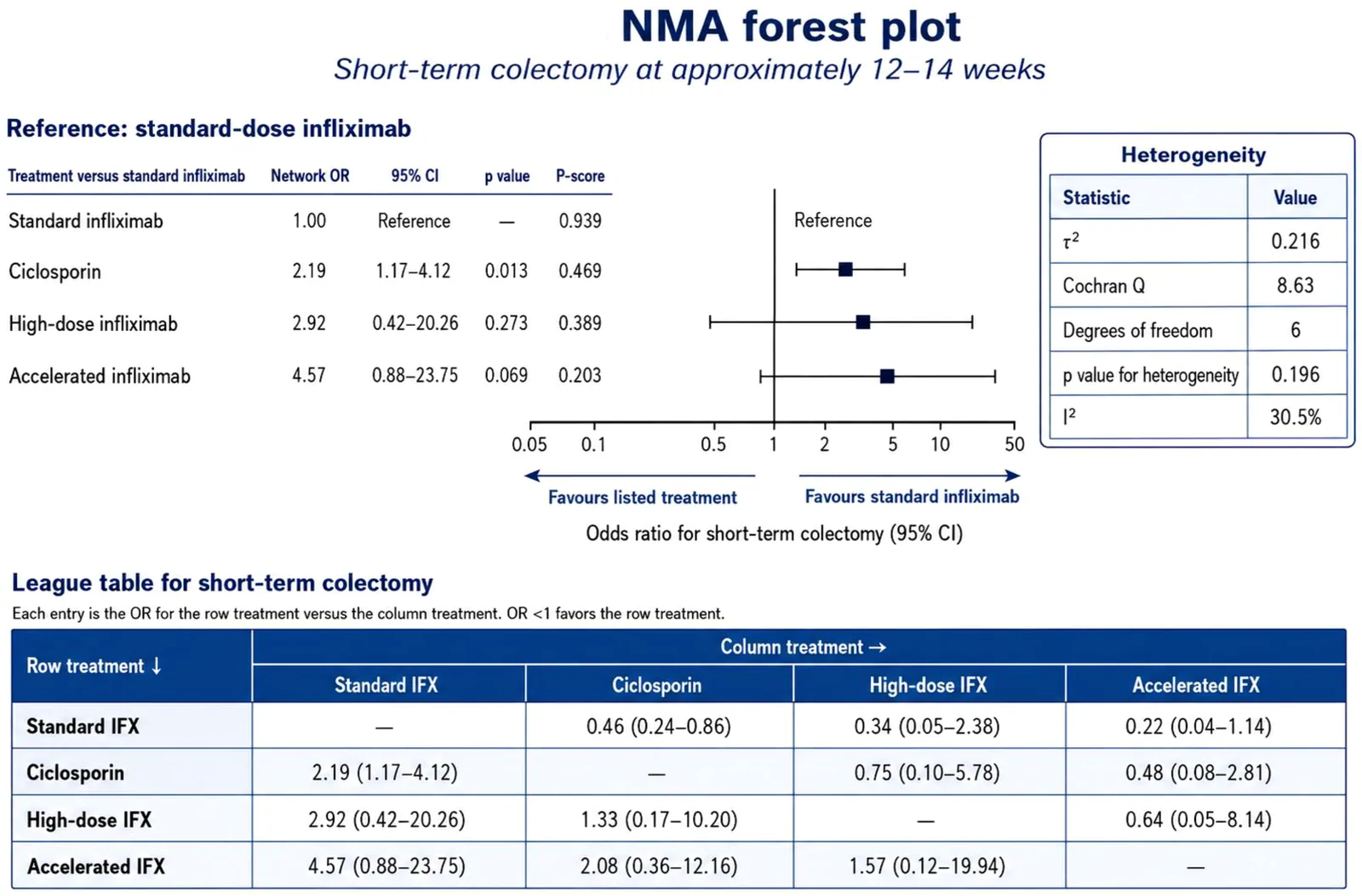
Network meta-analysis of short-term colectomy at approximately 12–14 weeks. Odds ratios greater than 1 indicate higher odds of colectomy relative to standard-dose infliximab. Estimates were generated using a random-effects frequentist network meta-analysis with restricted maximum-likelihood estimation of the common between-study variance. CI, confidence interval; IFX, infliximab.

#### 3.4.2 Early colectomy

Four studies involving 283 participants were included in the strict early-colectomy network, defined as colectomy during the index hospitalization or within 30 days. The network included ciclosporin, conventional infliximab, accelerated infliximab, and tacrolimus. No statistical heterogeneity was detected (τ²=0; I²=0%; Q = 0.83, p=0.362), although the small number of studies limited the power of this assessment.

Ciclosporin was associated with significantly higher odds of early colectomy than conventional infliximab (OR 3.05, 95% CI 1.38–6.77; p=0.006). Tacrolimus was not significantly different from conventional infliximab (OR 0.49, 95% CI 0.05–5.04). Accelerated infliximab was also not significantly different from conventional infliximab (OR 3.24, 95% CI 0.15–69.94), with extreme imprecision resulting from zero events in the conventional-infliximab group of the contributing study.

Tacrolimus had the highest numerical P-score (0.83), followed by conventional infliximab (0.68), accelerated infliximab (0.29), and ciclosporin (0.20). This ranking should not be interpreted as evidence that tacrolimus is superior because its network estimate was based on one retrospective study of 61 patients and had a very wide confidence interval. In that study, patients receiving tacrolimus had substantially greater previous exposure to advanced therapies, but colectomy-free response and safety were similar to infliximab.

The Mohamedrashed timing comparison constituted a separate two-node component. Early infliximab administration within 72 hours was not associated with lower in-hospital colectomy than later administration (OR 0.85, 95% CI 0.07–9.78). The study nevertheless reported a one-day reduction in median hospital stay with earlier infliximab treatment.

##### 3.4.2.1 Sensitivity analysis

Gibson et al. defined early colectomy over the broader infliximab induction period, which could extend beyond 30 days, and was therefore excluded from the strict primary window. When this study was included in a sensitivity analysis, the direction of the accelerated-infliximab estimate reversed, favoring accelerated treatment over conventional infliximab, although the difference remained nonsignificant (OR 0.35, 95% CI 0.05–2.30). Heterogeneity increased to approximately 50%. This marked change demonstrates that conclusions regarding accelerated induction were highly sensitive to the outcome window and the inclusion of individual observational studies (**Figures 5**, **6**).

**Figure 5:**
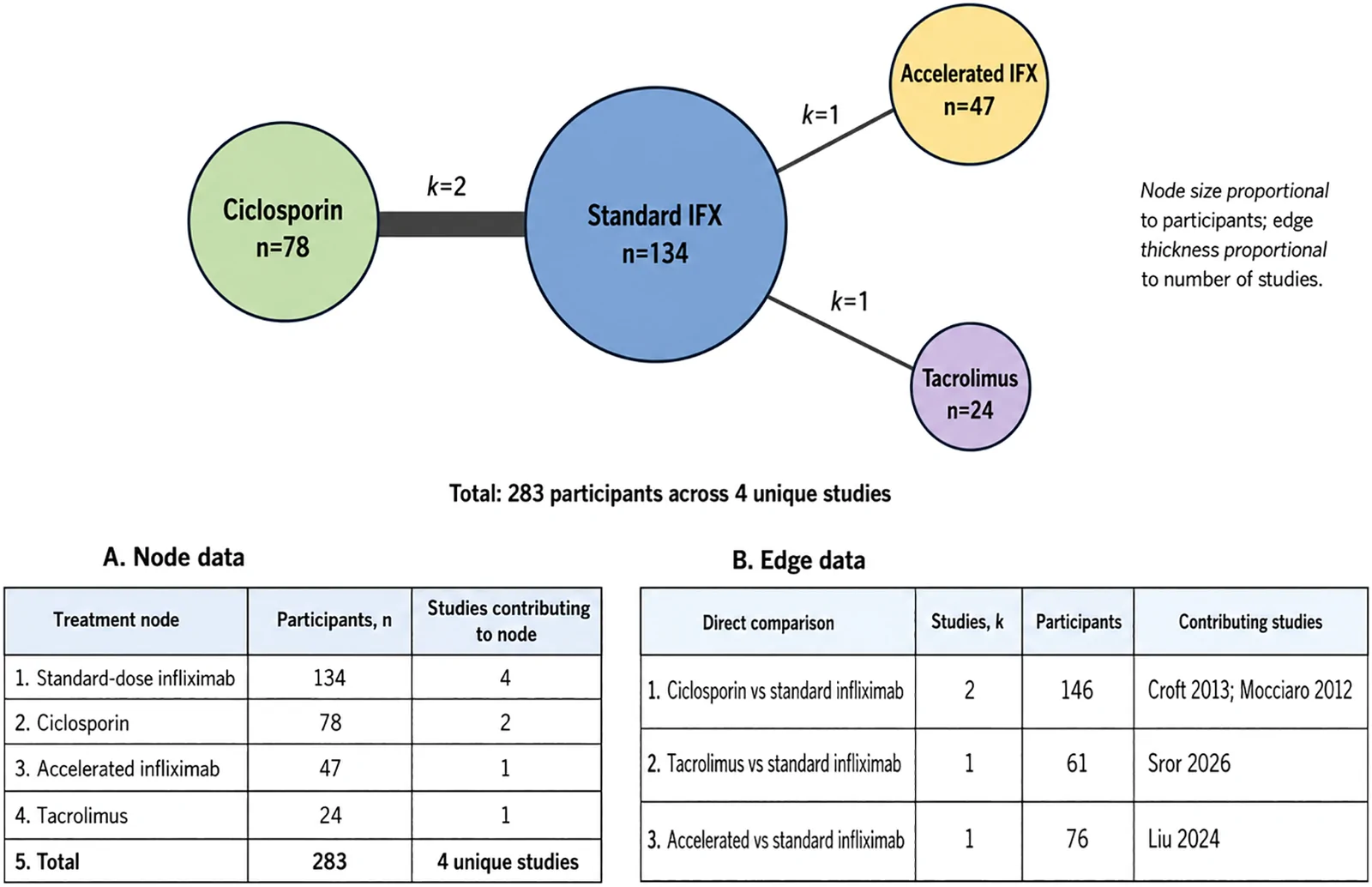
Network geometry for early colectomy during the index hospitalization or within 30 days. Node size is proportional to the number of participants and line thickness to the number of direct studies. Four studies involving 283 participants contributed to the connected network. No closed evidence loop was present.

**Figure 6:**
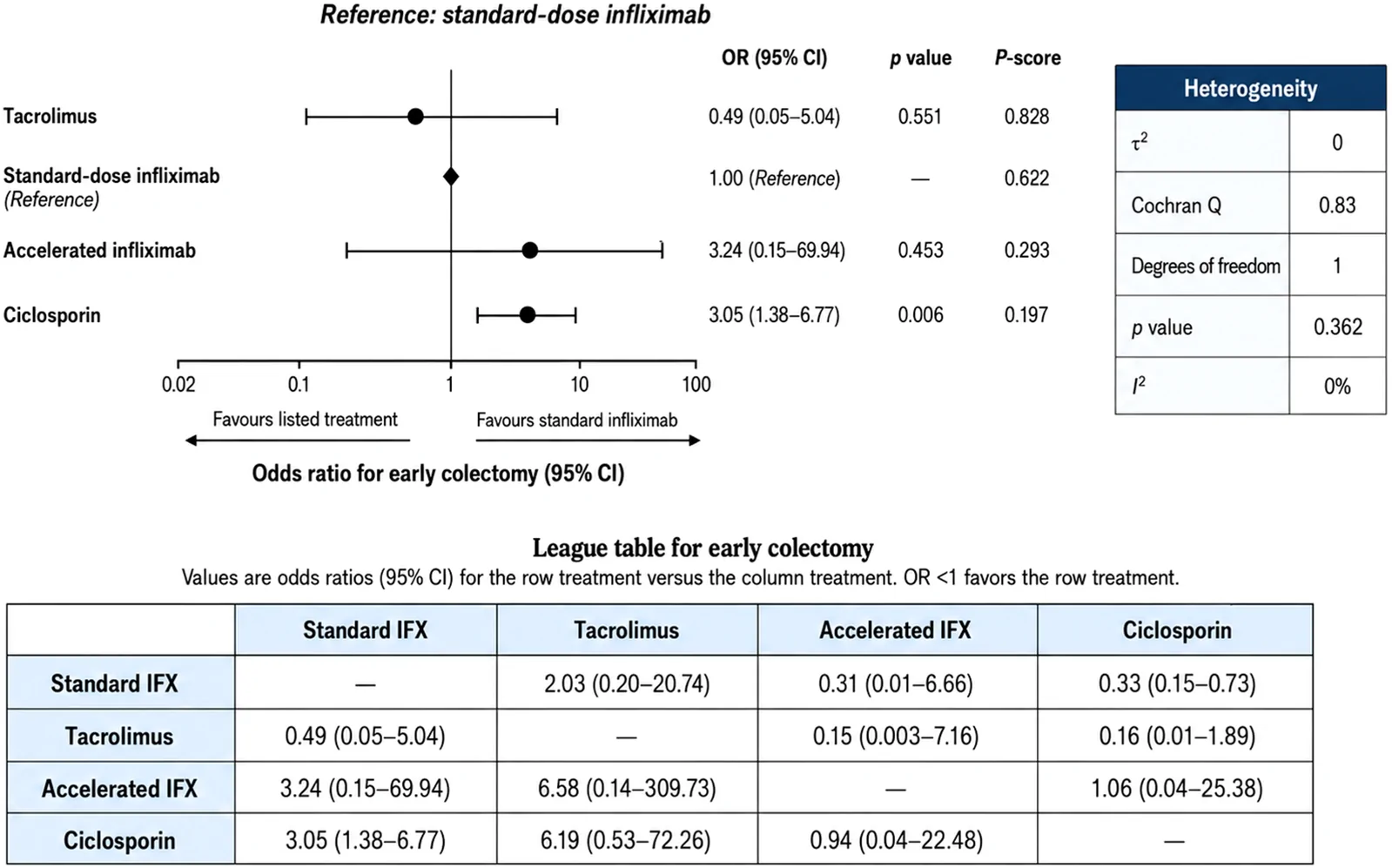
Network meta-analysis of early colectomy during hospitalization or within 30 days. Odds ratios greater than 1 indicate higher odds of early colectomy than with standard-dose infliximab. Treatment rankings should be interpreted cautiously because tacrolimus and accelerated infliximab were each informed by a single study.

#### 3.4.3 Colectomy at 12 months

Six studies involving 328 participants contributed to the 12-month network. Conventional infliximab, ciclosporin, accelerated infliximab, and tacrolimus were connected through direct comparisons with conventional infliximab. No measurable heterogeneity was identified (τ²=0; I²=0%; Q = 1.93, p=0.587).

Ciclosporin was associated with significantly higher odds of colectomy at approximately 12 months than conventional infliximab (OR 3.65, 95% CI 1.98–6.72; p<0.001). No significant difference was identified between tacrolimus and conventional infliximab (OR 1.17, 95% CI 0.23–5.84) or between accelerated and conventional infliximab (OR 2.22, 95% CI 0.56–8.76).

Conventional infliximab had the highest P-score for preventing 12-month colectomy (0.82), followed by tacrolimus (0.68), accelerated infliximab (0.38), and ciclosporin (0.12). Once again, the tacrolimus and accelerated-infliximab rankings were informed by single comparisons and should be considered exploratory.

The apparent long-term advantage of infliximab over ciclosporin should be interpreted in light of important differences in post-rescue management. In several historical cohorts, ciclosporin was used as a temporary bridge to thiopurine therapy, whereas infliximab could be continued as maintenance therapy. Treatment allocation was also closely related to calendar period in several studies. For example, Kim et al. reported substantially more concomitant azathioprine use in the infliximab group, and adjustment for confounding eliminated the statistically significant unadjusted association between rescue drug and colectomy-free survival.

Earlier versus later infliximab administration did not affect 12-month colectomy in the disconnected timing comparison (OR 1.03, 95% CI 0.10–10.73). This estimate was highly imprecise because long-term follow-up data were unavailable for a substantial proportion of participants.

For the JAK-inhibitor comparison, Honap et al. reported day-182 colectomy-free survival probabilities of 75% with tofacitinib and 78% with upadacitinib, without a significant difference (p=0.99). (**Figures 7**, **8**).

**Figure 7:**
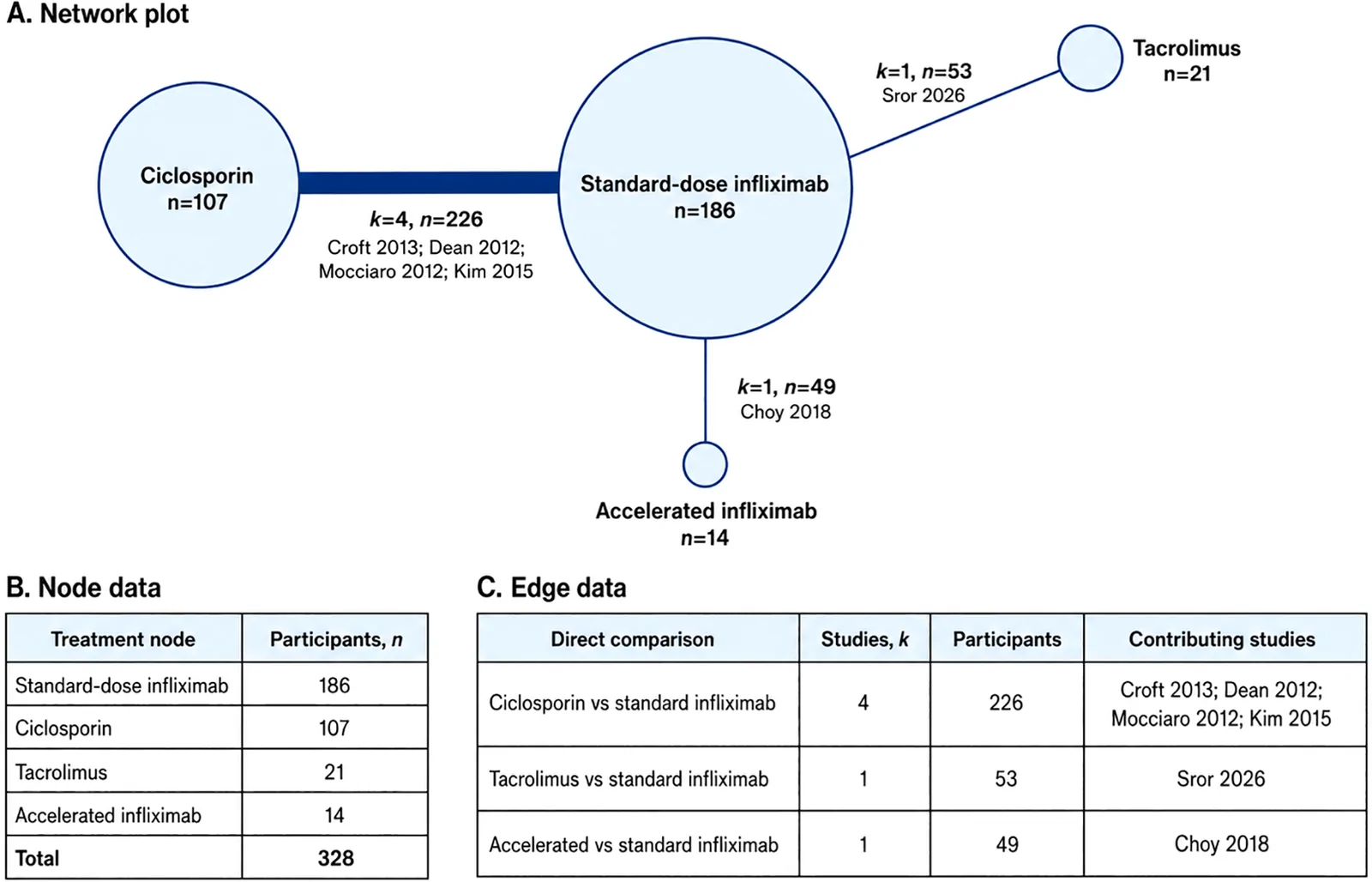
Network geometry for colectomy at approximately 12 months. Node size is proportional to the number of participants and line thickness to the number of studies contributing to each direct comparison. Six studies and 328 participants contributed to the connected network.

**Figure 8:**
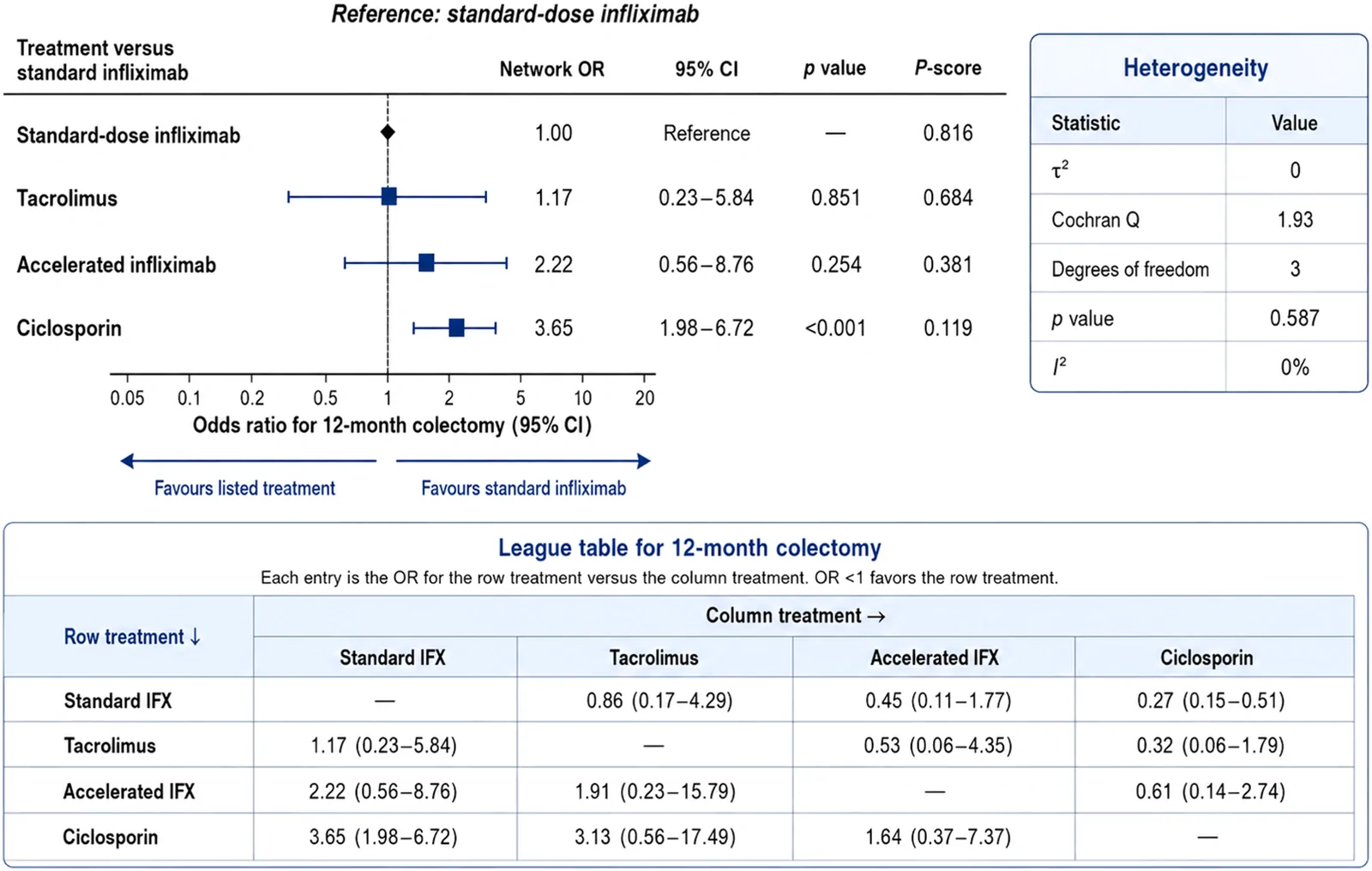
Network meta-analysis of colectomy at approximately 12 months. Odds ratios greater than 1 indicate higher odds of colectomy relative to standard-dose infliximab. Treatment rankings for tacrolimus and accelerated infliximab were based on single direct comparisons and should be considered exploratory.

#### 3.4.4 Serious adverse events

Four studies involving 509 participants provided comparative patient-level data for serious adverse events in the principal rescue-treatment network. There was no evidence of a difference between ciclosporin and infliximab (OR 0.94, 95% CI 0.54–1.63; p=0.819). Heterogeneity was minimal (τ²=0.001; I²=4.5%; Q = 3.14, p=0.370).

The randomized Laharie trial reported severe adverse events in 9 of 58 patients receiving ciclosporin and 14 of 57 receiving infliximab. The pragmatic CONSTRUCT trial similarly found no meaningful difference in patient-level serious adverse events between treatments. Croft et al. reported two serious adverse events, both seizures occurring during ciclosporin therapy, whereas Kim et al. reported one serious adverse event in each treatment group.

The JAK-inhibitor safety comparison was disconnected from the infliximab–ciclosporin network. Serious adverse events occurred in 14 of 60 patients receiving tofacitinib and 14 of 51 receiving upadacitinib, corresponding to an OR of 0.80 (95% CI 0.34–1.90). The original propensity-adjusted cohort similarly reported no significant safety difference between the two JAK inhibitors (**Figures 9**, **10**).

**Figure 9:**
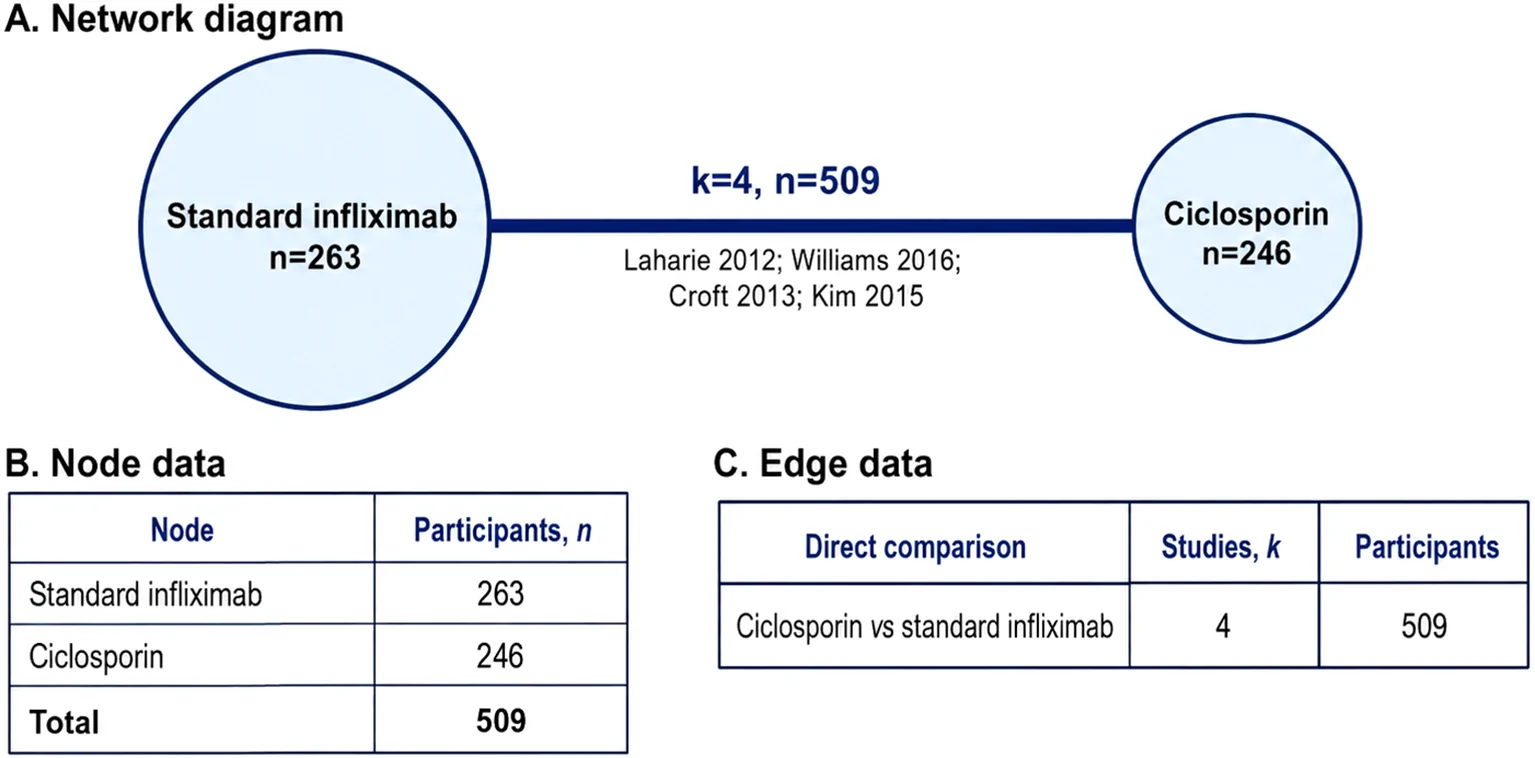
Network geometry for serious adverse events. Four studies involving 509 participants contributed to the comparative safety analysis. Node size represents the number of participants and line thickness represents the number of direct studies.

**Figure 10:**
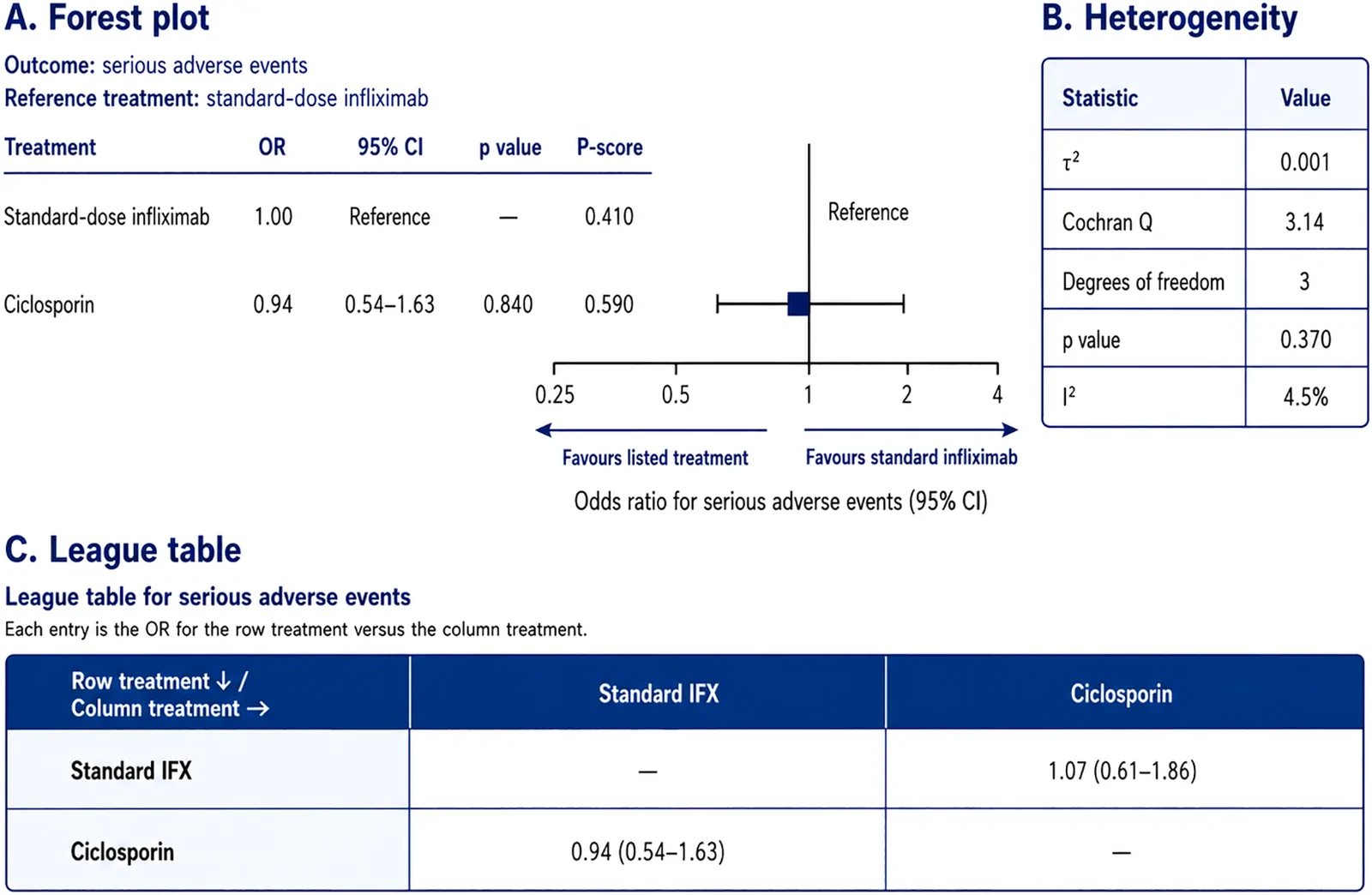
Comparative effect of ciclosporin and infliximab on serious adverse events. An odds ratio below 1 favors ciclosporin. No statistically significant difference in serious adverse events was identified between the treatments.

##### 3.4.4.1 Overall interpretation

Across the connected networks, conventional infliximab was associated with lower short-term, early, and 12-month colectomy odds than ciclosporin, while no significant safety difference was detected. However, this apparent efficacy advantage arose mainly from observational and historical-cohort evidence. The available randomized evidence did not demonstrate superiority of either infliximab or ciclosporin.

No consistent benefit was demonstrated for accelerated or high-dose infliximab over conventional infliximab. Estimates for tacrolimus, tofacitinib, and upadacitinib remained imprecise or disconnected from the conventional rescue-treatment network. Consequently, the rankings should be regarded as exploratory and should not be interpreted as a definitive hierarchy across all rescue therapies. A valid global NMA ranking biologic, calcineurin-inhibitor, and JAK-inhibitor treatments together was not possible with the available 18-study dataset.

### 3.5 GRADE assessment

The certainty of evidence was assessed for each principal treatment comparison using the Grading of Recommendations Assessment, Development and Evaluation framework adapted for network meta-analysis. Judgments considered risk of bias, inconsistency, indirectness, imprecision, publication bias, heterogeneity, and incoherence between direct and indirect evidence.

Overall, the certainty of evidence ranged from low to very low, and no comparison was supported by high- or moderate-certainty evidence. The main reasons for downgrading were the predominance of nonrandomized studies at serious risk of bias, residual confounding by indication, historical differences between treatment groups, small sample sizes, wide confidence intervals, and reliance on single-study treatment nodes.

For short-term colectomy, the comparison between ciclosporin and standard-dose infliximab was judged to provide low-certainty evidence. Although the network estimate favored standard-dose infliximab and its confidence interval excluded the null effect, the result was driven predominantly by observational studies at serious risk of bias. Moreover, the randomized comparison was neutral, whereas the pooled observational evidence favored infliximab, raising concerns regarding inconsistency between study designs.

Evidence comparing accelerated or high-dose infliximab with standard-dose infliximab was considered very low certainty. These comparisons were based on only one or two observational studies, were vulnerable to confounding by indication, and produced wide confidence intervals that included substantial benefit and substantial harm.

For early colectomy, the evidence suggesting higher colectomy odds with ciclosporin than with standard-dose infliximab was rated as very low certainty because the estimate was based on only two observational studies at serious risk of bias. Comparisons involving tacrolimus and accelerated infliximab were also rated as very low certainty because each was informed by a single retrospective study and had extremely imprecise estimates.

The evidence for colectomy at approximately 12 months was similarly rated as very low certainty for all efficacy comparisons. The apparent advantage of standard-dose infliximab over ciclosporin was derived exclusively from observational cohorts that were susceptible to confounding, differences in treatment era, and differences in post-rescue maintenance therapy. Estimates involving tacrolimus and accelerated infliximab were based on single studies with wide confidence intervals.

The comparison of ciclosporin and infliximab for serious adverse events was supported by low-certainty evidence. The inclusion of randomized trials and the minimal statistical heterogeneity increased confidence in this estimate; however, the confidence interval remained compatible with clinically important benefit or harm in either direction, and some contributing evidence was obtained from nonrandomized studies.

Formal assessment of incoherence was not possible because none of the networks contained a closed evidence loop. This limitation was considered when interpreting the certainty of indirect comparisons but was not treated automatically as evidence of incoherence. Evidence involving tofacitinib and upadacitinib was not incorporated into the principal GRADE network assessment because these comparisons were disconnected from the main network. The certainty of the available direct JAK-inhibitor evidence was considered very low because it originated from retrospective studies with substantial potential for residual confounding. (**Table 3**).

**Table 3:** Summary of findings and certainty of evidence according to the GRADE framework for major efficacy and safety outcomes comparing intracardiac echocardiography (ICE) versus transesophageal echocardiography (TEE) in patients undergoing catheter ablation for atrial fibrillation.

Outcome and comparison	Evidence base	Network effect, OR (95% CI)	Main certainty concerns	Certainty of evidence	Interpretation
Short-term colectomy: ciclosporin vs standard-dose infliximab	6 studies; 462 participants	2.19 (1.17–4.12)	Serious risk of bias; divergence between randomized and observational findings	Low ⊕⊕○○	Ciclosporin may increase short-term colectomy compared with standard-dose infliximab
Short-term colectomy: accelerated vs standard-dose infliximab	2 studies; 123 participants	4.57 (0.88–23.75)	Serious risk of bias; very serious imprecision; small treatment node	Very low ⊕○○○	The effect of accelerated infliximab is very uncertain
Short-term colectomy: high-dose vs standard-dose infliximab	1 study; 72 participants	2.92 (0.42–20.26)	Serious risk of bias; very serious imprecision; single-study estimate	Very low ⊕○○○	The effect of high-dose infliximab is very uncertain
Early colectomy: ciclosporin vs standard-dose infliximab	2 studies; 146 participants	3.05 (1.38–6.77)	Observational evidence only; serious confounding and risk of bias; limited sample size	Very low ⊕○○○	Ciclosporin may increase early colectomy, but the evidence is highly uncertain
Early colectomy: tacrolimus vs standard-dose infliximab	1 study; 61 participants	0.49 (0.05–5.04)	Serious risk of bias; very serious imprecision; single-study estimate	Very low ⊕○○○	The comparative effect of tacrolimus is very uncertain
Early colectomy: accelerated vs standard-dose infliximab	1 study; 76 participants	3.24 (0.15–69.94)	Serious risk of bias; extremely serious imprecision; sparse events	Very low ⊕○○○	The comparative effect of accelerated infliximab is very uncertain
Twelve-month colectomy: ciclosporin vs standard-dose infliximab	4 studies; 226 participants	3.65 (1.98–6.72)	Observational evidence only; serious confounding; treatment-era and maintenance-strategy differences	Very low ⊕○○○	Ciclosporin may increase 12-month colectomy, but confidence in the estimate is very limited
Twelve-month colectomy: tacrolimus vs standard-dose infliximab	1 study; 53 participants	1.17 (0.23–5.84)	Serious risk of bias; very serious imprecision; single-study estimate	Very low ⊕○○○	The comparative effect of tacrolimus is very uncertain
Twelve-month colectomy: accelerated vs standard-dose infliximab	1 study; 49 participants	2.22 (0.56–8.76)	Serious risk of bias; very serious imprecision; single-study estimate	Very low ⊕○○○	The comparative effect of accelerated infliximab is very uncertain
Serious adverse events: ciclosporin vs standard-dose infliximab	4 studies; 509 participants	0.94 (0.54–1.63)	Study limitations and serious imprecision; confidence interval includes meaningful benefit and harm	Low ⊕⊕○○	Ciclosporin and infliximab may have similar serious-adverse-event rates

## 4 Discussion

This systematic review and network meta-analysis compared the efficacy and safety of medical rescue strategies for steroid-refractory acute severe ulcerative colitis. Eighteen comparative studies involving 1, 529 participants were included, comprising three randomized controlled trials and 15 observational studies. Separate connected networks were evaluated for early colectomy during hospitalization or within 30 days, short-term colectomy at approximately 12–14 weeks, colectomy at approximately 12 months, and serious adverse events.

Across the combined randomized and observational evidence, standard-dose infliximab was associated with lower odds of colectomy than ciclosporin at early, short-term, and 12-month follow-up. Ciclosporin was associated with approximately three-fold higher odds of early colectomy, more than two-fold higher odds of short-term colectomy, and more than three-fold higher odds of colectomy at approximately 12 months. In contrast, no statistically significant differences were found between standard-dose infliximab and tacrolimus, accelerated infliximab, or high-dose infliximab. Serious adverse events were also comparable between infliximab and ciclosporin.

These findings require cautious interpretation. The apparent advantage of infliximab over ciclosporin was driven predominantly by nonrandomized and historical-cohort evidence, whereas the major randomized trials did not demonstrate the superiority of either treatment. Furthermore, the networks contained no closed loops, preventing formal assessment of inconsistency between direct and indirect evidence. Several treatment nodes were informed by only one study, resulting in wide confidence intervals and unstable treatment rankings.

The apparent advantage of standard-dose infliximab in the combined network differs from the results of the principal randomized trials. In the CySIF trial, Laharie et al. found no significant difference between infliximab and ciclosporin in treatment failure, colectomy, clinical response, mucosal healing, or serious adverse events at 98 days (5). Similarly, the pragmatic CONSTRUCT trial found no meaningful difference in colectomy, mortality, serious adverse events, quality-adjusted survival, or overall clinical effectiveness between the two rescue strategies (6). Accordingly, current guidelines continue to recognize both infliximab and ciclosporin as appropriate rescue treatments for patients who fail to respond adequately to intravenous corticosteroids (3, 4).

Previous evidence syntheses have reported a similar difference between randomized and observational evidence. Narula et al. found no convincing difference between infliximab and ciclosporin in randomized comparisons, whereas nonrandomized studies generally favored infliximab for short- and long-term colectomy avoidance (42). Barberio et al. also concluded that randomized evidence did not establish the superiority of either active rescue therapy (7). The more recent network meta-analysis by Huang et al. included additional treatment strategies but similarly emphasized the limited certainty of comparisons based primarily on observational evidence (11).

Several factors may explain why observational studies favored infliximab. Many studies used historical treatment groups in which ciclosporin was administered during earlier periods and infliximab during more contemporary periods (30, 31, 33, 35, 38). Improvements over time in supportive care, venous thromboembolism prophylaxis, infection screening, nutritional support, multidisciplinary decision-making, and surgical management may therefore have improved outcomes in the later infliximab cohorts independently of the rescue drug itself.

Differences in post-rescue maintenance treatment may also have influenced longer-term colectomy rates. Infliximab could generally be continued as maintenance therapy after successful induction, whereas ciclosporin was commonly used as a temporary bridge to thiopurines (30, 35, 37). Patients who had previously failed or were intolerant of thiopurines may consequently have had fewer effective maintenance options after ciclosporin. The apparent 12-month benefit of infliximab may therefore reflect differences in the complete induction and maintenance strategy rather than the isolated pharmacological effect of the initial rescue therapy.

Treatment selection in observational practice was also influenced by age, disease severity, inflammatory burden, serum albumin, previous biologic exposure, comorbidities, clinician preference, and institutional experience. Residual confounding by indication is therefore likely, even in studies that used multivariable adjustment, matching, or propensity-score methods.

The present findings consequently support standard-dose infliximab as an effective and practical rescue treatment but do not establish its definitive superiority over ciclosporin. Ciclosporin remains an evidence-based option, particularly in patients with previous anti-TNF failure, in thiopurine-naïve patients, when infliximab is contraindicated, or in centers with experience in therapeutic drug monitoring and ciclosporin-to-maintenance transition strategies.

The optimal infliximab induction regimen in steroid-refractory ASUC remains uncertain. Intensified induction has a biological rationale because severe intestinal inflammation, hypoalbuminemia, fecal drug loss, and increased systemic clearance may reduce infliximab exposure. Early observational evidence suggested that accelerated induction might reduce colectomy during the induction period (32). However, subsequent studies did not consistently reproduce this benefit (28, 29, 34, 40).

In the present analysis, neither accelerated nor high-dose infliximab was significantly different from standard-dose infliximab for short-term colectomy. Accelerated infliximab was also not associated with significantly different early or 12-month colectomy odds. The confidence intervals were wide, reflecting small treatment groups, low event counts, and the limited number of direct comparisons.

Interpretation is further complicated by confounding by indication. Patients receiving accelerated or high-dose infliximab frequently had more severe disease, lower serum albumin, higher inflammatory markers, or inadequate early responses to standard treatment. Such baseline differences may make intensified regimens appear less effective, even when escalation is selected for patients with a worse initial prognosis.

The PREDICT-UC randomized trial provides the most reliable contemporary evidence regarding infliximab intensification. An initial infliximab dose of 10 mg/kg was not superior to 5 mg/kg for clinical response by day 7, and no significant differences were identified among intensified, accelerated, and standard induction strategies for remission or colectomy by 3 months (8). These findings indicate that routine intensified induction should not be assumed to improve outcomes for all patients with steroid-refractory ASUC.

Nevertheless, intensified treatment may remain appropriate in selected patients with profound hypoalbuminemia, high inflammatory burden, suspected rapid infliximab clearance, or inadequate biochemical response after the first infusion. Future studies should evaluate pharmacokinetic- or biomarker-guided treatment algorithms rather than applying accelerated or high-dose induction uniformly.

Tacrolimus was represented by one retrospective comparative study involving 61 patients (39). No statistically significant difference was identified between tacrolimus and standard-dose infliximab for early or 12-month colectomy. Although tacrolimus had the highest numerical P-score in the early-colectomy network, its estimate was based on only 24 tacrolimus-treated patients and was accompanied by a very wide confidence interval.

The tacrolimus group also had substantially greater previous exposure to anti-TNF agents and other advanced therapies than the infliximab group, indicating a more treatment-refractory population. The absence of a clear difference despite these baseline imbalances is clinically relevant but should not be interpreted as evidence of tacrolimus superiority.

Tacrolimus may represent a useful rescue option in experienced centers, particularly when infliximab has previously failed or is contraindicated. However, prospective comparative studies with standardized target drug concentrations, clearly defined corticosteroid failure, and consistent maintenance strategies are required before its relative position can be determined reliably.

The evidence for tofacitinib and upadacitinib could not be incorporated into the principal connected networks because the available studies lacked a sufficiently comparable common treatment node.

Berinstein et al. reported that tofacitinib was associated with a lower adjusted 90-day colectomy risk than matched standard-care controls in biologic-experienced hospitalized patients (9). The apparent benefit was strongest among patients receiving tofacitinib 10 mg three times daily. However, all tofacitinib-treated patients had previous biologic exposure, the comparator group was clinically heterogeneous, and treatment was administered alongside intravenous corticosteroids. Residual confounding and differences in baseline treatment history therefore remain important limitations.

Honap et al. directly compared tofacitinib with upadacitinib in an international retrospective cohort (10). Upadacitinib was associated with a more rapid early clinical response, but later clinical outcomes, colectomy-free survival, and safety were similar between treatments. In the strict salvage subgroup, colectomy-free survival remained almost identical at approximately 14 and 26 weeks.

The rapid onset of action, oral administration, and short half-life of JAK inhibitors make them attractive rescue options, especially in patients with previous anti-TNF exposure. Nevertheless, the available evidence is observational and includes variation in the timing and indication for treatment. Some patients received a JAK inhibitor after documented corticosteroid failure, whereas others received treatment before strict steroid refractoriness had been established. Randomized studies in clearly defined steroid-refractory populations are therefore needed before JAK inhibitors can be reliably compared with infliximab, ciclosporin, tacrolimus, or surgery.

No statistically significant difference in serious adverse events was identified between ciclosporin and infliximab. This finding is consistent with the CySIF and CONSTRUCT randomized trials, which did not demonstrate a clear overall safety advantage for either rescue strategy (5, 6). Comparative observational studies also reported few serious events, although individual toxicities differed between therapies (30, 33).

Ciclosporin requires therapeutic drug monitoring and is associated with risks of nephrotoxicity, hypertension, electrolyte abnormalities, seizures, opportunistic infection, and drug interactions. Infliximab is associated with infusion reactions, infection, reactivation of latent infections, immunogenicity, and loss of response. Similar aggregate serious-adverse-event rates therefore do not imply identical safety profiles.

No significant difference in serious adverse events was found between tofacitinib and upadacitinib (10). However, the number of safety events and duration of follow-up were insufficient to assess uncommon outcomes such as venous thromboembolism, herpes zoster, major cardiovascular events, malignancy, or serious opportunistic infection.

### 4.1 Clinical implications

The findings support standard-dose infliximab as a reasonable default rescue strategy for many patients with steroid-refractory ASUC. Its practical advantages include established efficacy, clinician familiarity, and the ability to continue the same treatment for maintenance. However, the randomized evidence supports broadly comparable short-term efficacy between infliximab and ciclosporin, and ciclosporin should not be regarded as an ineffective or obsolete treatment (3–7).

Treatment selection should be individualized according to previous biologic and thiopurine exposure, serum albumin, inflammatory burden, comorbidities, contraindications, infection risk, maintenance options, patient preference, and institutional expertise. Tacrolimus and JAK inhibitors may be considered in selected patients, particularly after previous anti-TNF exposure, but the comparative evidence remains limited.

Early colorectal surgical consultation is essential and should occur in parallel with medical rescue planning. Treatment rankings derived from sparse networks should not justify repeated ineffective medical rescue attempts or delay colectomy in patients with clinical deterioration, toxic megacolon, uncontrolled bleeding, perforation, or failure to improve promptly.

### 4.2 Strengths and limitations

This review has several strengths. It incorporated both randomized and comparative real-world evidence, evaluated clinically distinct colectomy time points, and separated alternative infliximab dosing and timing strategies from comparisons between different drug classes. The search included five major electronic databases, abstract-only reports were excluded, and risk of bias was assessed using design-specific tools. Network geometry plots, forest plots, league tables, and P-scores provided a transparent presentation of the available evidence.

Several limitations must also be acknowledged. First, most included studies were observational, and 14 of the 15 nonrandomized studies were judged to have a serious overall risk of bias. Confounding by indication was particularly important because patients with more severe or treatment-refractory disease were more likely to receive intensified infliximab, tacrolimus, or JAK inhibitors.

Second, randomized and observational studies were synthesized within the same networks. Although this increased network connectivity, it also allowed nonrandomized evidence to influence the pooled estimates substantially. The observed advantage of infliximab over ciclosporin was largely driven by observational studies, whereas the randomized trials were neutral.

Third, the networks did not contain closed loops. Formal node-splitting and loop-specific inconsistency analyses could therefore not be undertaken. All indirect comparisons depended on standard-dose infliximab as the common comparator.

Fourth, definitions of steroid failure, accelerated infliximab treatment, serious adverse events, and outcome follow-up windows varied across studies. Some studies assessed colectomy during hospitalization, whereas others used 30-day, induction-period, 90-day, or 12-month definitions.

Fifth, treatment-era effects and differences in maintenance therapy may have influenced longer-term outcomes. Several ciclosporin cohorts were treated during earlier periods and transitioned to thiopurines, whereas infliximab could be continued for maintenance.

Sixth, the tacrolimus, accelerated-infliximab, and high-dose-infliximab nodes were informed by only one or two direct comparisons. Their estimates were therefore imprecise, and the corresponding P-scores should be considered exploratory.

Although major potential effect modifiers were examined when assessing the plausibility of transitivity, transitivity cannot be established empirically in a sparse network. Residual differences between randomized and observational populations, particularly in treatment era, baseline disease severity, previous advanced-therapy exposure, rescue-treatment selection, and post-rescue maintenance strategies, may have influenced the pooled estimates. Study-design-specific subgroup analyses and meta-regression were not feasible because the number of studies within individual treatment contrasts was insufficient and several nodes were informed by only a single study. Accordingly, the combined network estimates should be interpreted as an integration of the available comparative evidence rather than as definitive evidence that randomized and observational studies were fully exchangeable.

Dedicated grey-literature sources and conference proceedings were not systematically searched, and abstract-only reports were excluded. Therefore, publication and dissemination bias cannot be completely excluded.

Finally, tofacitinib and upadacitinib could not be connected validly to the principal rescue-treatment network. A global ranking incorporating biologic therapies, calcineurin inhibitors, infliximab regimens, and JAK inhibitors was therefore not possible.

### 4.3 Recommendations for future research

Future randomized trials should enroll patients with clearly documented corticosteroid-refractory ASUC and stratify randomization according to previous biologic or small-molecule exposure. Priority comparisons include standard-dose infliximab versus biomarker-guided infliximab intensification, infliximab versus JAK inhibitors, and comparative rescue strategies among patients with previous anti-TNF failure.

Studies should use standardized definitions and report colectomy during hospitalization, at 30 days, at approximately 90 days, and at 12 months. Time-to-colectomy estimates, clinical response, corticosteroid-free remission, length of hospitalization, readmission, serious infection, thromboembolism, postoperative complications, and mortality should also be reported consistently.

Prospective pharmacokinetic studies integrating serum albumin, C-reactive protein, infliximab clearance, fecal drug loss, and early biochemical response may identify patients who could benefit from individualized infliximab dosing. Long-term studies should also account for differences in maintenance therapy when comparing rescue strategies.

## 5 Conclusion

In this network meta-analysis, standard-dose infliximab was associated with lower odds of early, short-term, and 12-month colectomy than ciclosporin, while serious adverse-event rates were similar. No consistent benefit was identified for accelerated or high-dose infliximab, and estimates for tacrolimus and JAK inhibitors remained sparse or disconnected from the principal network. The apparent efficacy advantage of infliximab was driven mainly by observational studies, whereas randomized trials demonstrated comparable outcomes between infliximab and ciclosporin. The results therefore support standard infliximab as an effective and practical rescue strategy but do not establish its definitive superiority. Rescue-treatment selection should remain individualized according to previous therapy, disease severity, comorbidities, maintenance options, and institutional experience, with early surgical involvement and avoidance of delayed colectomy.

## Data Availability

The original contributions presented in the study are included in the article/**Supplementary Material**. Further inquiries can be directed to the corresponding author.
