# Supplementary material for: Comparative outcomes of advanced medical rescue therapies in steroid-refractory acute severe ulcerative colitis: a systematic review and network meta-analysis
Source: Front Gastroenterol (Lausanne). 2026 Sep 18;5:1937925. doi: 10.3389/fgstr.2026.1937925 (PMC13630618; doi:10.3389/fgstr.2026.1937925)
Supplement: Supplementary file 1 [file Table1.docx]

# 1. Search and Screening

*Appendix Table A1. Detailed Search Strategy Across Databases*

| **Database/platform** | **Complete search strategy** |
| --- | --- |
| MEDLINE via PubMed | ("Colitis, Ulcerative"[Mesh] OR "ulcerative colitis"[tiab] OR "ulcerative pancolitis"[tiab] OR "ulcerative colitides"[tiab])  AND  ("acute severe ulcerative colitis"[tiab] OR "acute severe colitis"[tiab] OR ASUC[tiab] OR "SR-ASUC"[tiab] OR "SR ASUC"[tiab] OR "steroid refractory"[tiab] OR "steroid-refractory"[tiab] OR "steroid resistant"[tiab] OR "steroid-resistant"[tiab] OR "corticosteroid refractory"[tiab] OR "corticosteroid-refractory"[tiab] OR "corticosteroid resistant"[tiab] OR "corticosteroid-resistant"[tiab] OR "steroid failure"[tiab] OR "corticosteroid failure"[tiab] OR "steroid nonresponse"[tiab] OR "steroid non-response"[tiab] OR "corticosteroid nonresponse"[tiab] OR "corticosteroid non-response"[tiab] OR "intravenous steroid failure"[tiab] OR "intravenous corticosteroid failure"[tiab] OR "fulminant colitis"[tiab] OR "severe colitis"[tiab] OR "rescue therapy"[tiab] OR "rescue treatment"[tiab] OR "medical rescue"[tiab] OR "salvage therapy"[tiab] OR "salvage treatment"[tiab] OR "sequential rescue"[tiab] OR "second-line rescue"[tiab] OR "second line rescue"[tiab] OR "Truelove and Witts"[tiab] OR "Oxford criteria"[tiab])  AND  ("Infliximab"[Mesh] OR "Cyclosporine"[Mesh] OR "Tacrolimus"[Mesh] OR "Tumor Necrosis Factor Inhibitors"[Mesh] OR "Janus Kinase Inhibitors"[Mesh] OR infliximab[tiab] OR Remicade[tiab] OR Remsima[tiab] OR Inflectra[tiab] OR "CT-P13"[tiab] OR cyclosporine[tiab] OR cyclosporin[tiab] OR ciclosporin[tiab] OR ciclosporine[tiab] OR tacrolimus[tiab] OR FK506[tiab] OR tofacitinib[tiab] OR Xeljanz[tiab] OR upadacitinib[tiab] OR Rinvoq[tiab] OR filgotinib[tiab] OR vedolizumab[tiab] OR ustekinumab[tiab] OR adalimumab[tiab] OR golimumab[tiab] OR "anti-TNF"[tiab] OR "anti TNF"[tiab] OR "TNF inhibitor"[tiab] OR "TNF inhibitors"[tiab] OR "JAK inhibitor"[tiab] OR "JAK inhibitors"[tiab] OR "Janus kinase inhibitor"[tiab] OR "Janus kinase inhibitors"[tiab] OR "calcineurin inhibitor"[tiab] OR "calcineurin inhibitors"[tiab] OR biologic*[tiab] OR "biological therapy"[tiab] OR "biological therapies"[tiab] OR "small molecule"[tiab] OR "small molecules"[tiab] OR "advanced therapy"[tiab] OR "advanced therapies"[tiab] OR "accelerated infliximab"[tiab] OR "accelerated-dose infliximab"[tiab] OR "accelerated dose infliximab"[tiab] OR "intensified infliximab"[tiab] OR "high-dose infliximab"[tiab] OR "high dose infliximab"[tiab]) |
| Embase via Embase.com | ('ulcerative colitis'/exp OR 'ulcerative colitis':ti,ab,kw OR 'ulcerative pancolitis':ti,ab,kw OR 'ulcerative colitides':ti,ab,kw)  AND  ('acute severe ulcerative colitis':ti,ab,kw OR 'acute severe colitis':ti,ab,kw OR asuc:ti,ab,kw OR 'sr-asuc':ti,ab,kw OR 'sr asuc':ti,ab,kw OR 'steroid refractory':ti,ab,kw OR 'steroid-refractory':ti,ab,kw OR 'steroid resistant':ti,ab,kw OR 'steroid-resistant':ti,ab,kw OR 'corticosteroid refractory':ti,ab,kw OR 'corticosteroid-refractory':ti,ab,kw OR 'corticosteroid resistant':ti,ab,kw OR 'corticosteroid-resistant':ti,ab,kw OR 'steroid failure':ti,ab,kw OR 'corticosteroid failure':ti,ab,kw OR 'steroid nonresponse':ti,ab,kw OR 'steroid non-response':ti,ab,kw OR 'corticosteroid nonresponse':ti,ab,kw OR 'corticosteroid non-response':ti,ab,kw OR 'intravenous steroid failure':ti,ab,kw OR 'intravenous corticosteroid failure':ti,ab,kw OR 'fulminant colitis':ti,ab,kw OR 'severe colitis':ti,ab,kw OR 'rescue therapy':ti,ab,kw OR 'rescue treatment':ti,ab,kw OR 'medical rescue':ti,ab,kw OR 'salvage therapy':ti,ab,kw OR 'salvage treatment':ti,ab,kw OR 'sequential rescue':ti,ab,kw OR 'second-line rescue':ti,ab,kw OR 'second line rescue':ti,ab,kw OR 'truelove and witts':ti,ab,kw OR 'oxford criteria':ti,ab,kw)  AND  ('infliximab'/exp OR 'tacrolimus'/exp OR 'tofacitinib'/exp OR 'upadacitinib'/exp OR infliximab:ti,ab,kw OR remicade:ti,ab,kw OR remsima:ti,ab,kw OR inflectra:ti,ab,kw OR 'ct-p13':ti,ab,kw OR cyclosporine:ti,ab,kw OR cyclosporin:ti,ab,kw OR ciclosporin:ti,ab,kw OR ciclosporine:ti,ab,kw OR tacrolimus:ti,ab,kw OR fk506:ti,ab,kw OR tofacitinib:ti,ab,kw OR xeljanz:ti,ab,kw OR upadacitinib:ti,ab,kw OR rinvoq:ti,ab,kw OR filgotinib:ti,ab,kw OR vedolizumab:ti,ab,kw OR ustekinumab:ti,ab,kw OR adalimumab:ti,ab,kw OR golimumab:ti,ab,kw OR 'anti-tnf':ti,ab,kw OR 'anti tnf':ti,ab,kw OR 'tnf inhibitor':ti,ab,kw OR 'tnf inhibitors':ti,ab,kw OR 'jak inhibitor':ti,ab,kw OR 'jak inhibitors':ti,ab,kw OR 'janus kinase inhibitor':ti,ab,kw OR 'janus kinase inhibitors':ti,ab,kw OR 'calcineurin inhibitor':ti,ab,kw OR 'calcineurin inhibitors':ti,ab,kw OR biologic*:ti,ab,kw OR 'biological therapy':ti,ab,kw OR 'biological therapies':ti,ab,kw OR 'small molecule':ti,ab,kw OR 'small molecules':ti,ab,kw OR 'advanced therapy':ti,ab,kw OR 'advanced therapies':ti,ab,kw OR 'accelerated infliximab':ti,ab,kw OR 'accelerated-dose infliximab':ti,ab,kw OR 'accelerated dose infliximab':ti,ab,kw OR 'intensified infliximab':ti,ab,kw OR 'high-dose infliximab':ti,ab,kw OR 'high dose infliximab':ti,ab,kw) |
| Scopus | TITLE-ABS-KEY(  ("ulcerative colitis" OR "ulcerative pancolitis" OR "ulcerative colitides")  AND  ("acute severe ulcerative colitis" OR "acute severe colitis" OR ASUC OR "SR-ASUC" OR "SR ASUC" OR "steroid refractory" OR "steroid-refractory" OR "steroid resistant" OR "steroid-resistant" OR "corticosteroid refractory" OR "corticosteroid-refractory" OR "corticosteroid resistant" OR "corticosteroid-resistant" OR "steroid failure" OR "corticosteroid failure" OR "steroid nonresponse" OR "steroid non-response" OR "corticosteroid nonresponse" OR "corticosteroid non-response" OR "intravenous steroid failure" OR "intravenous corticosteroid failure" OR "fulminant colitis" OR "severe colitis" OR "rescue therapy" OR "rescue treatment" OR "medical rescue" OR "salvage therapy" OR "salvage treatment" OR "sequential rescue" OR "second-line rescue" OR "second line rescue" OR "Truelove and Witts" OR "Oxford criteria")  AND  (infliximab OR Remicade OR Remsima OR Inflectra OR "CT-P13" OR cyclosporine OR cyclosporin OR ciclosporin OR ciclosporine OR tacrolimus OR FK506 OR tofacitinib OR Xeljanz OR upadacitinib OR Rinvoq OR filgotinib OR vedolizumab OR ustekinumab OR adalimumab OR golimumab OR "anti-TNF" OR "anti TNF" OR "TNF inhibitor" OR "TNF inhibitors" OR "JAK inhibitor" OR "JAK inhibitors" OR "Janus kinase inhibitor" OR "Janus kinase inhibitors" OR "calcineurin inhibitor" OR "calcineurin inhibitors" OR biologic* OR "biological therapy" OR "biological therapies" OR "small molecule" OR "small molecules" OR "advanced therapy" OR "advanced therapies" OR "accelerated infliximab" OR "accelerated-dose infliximab" OR "accelerated dose infliximab" OR "intensified infliximab" OR "high-dose infliximab" OR "high dose infliximab")  ) |
| Web of Science Core Collection | ("ulcerative colitis" OR "ulcerative pancolitis" OR "ulcerative colitides")  AND  ("acute severe ulcerative colitis" OR "acute severe colitis" OR ASUC OR "SR-ASUC" OR "SR ASUC" OR "steroid refractory" OR "steroid-refractory" OR "steroid resistant" OR "steroid-resistant" OR "corticosteroid refractory" OR "corticosteroid-refractory" OR "corticosteroid resistant" OR "corticosteroid-resistant" OR "steroid failure" OR "corticosteroid failure" OR "steroid nonresponse" OR "steroid non-response" OR "corticosteroid nonresponse" OR "corticosteroid non-response" OR "intravenous steroid failure" OR "intravenous corticosteroid failure" OR "fulminant colitis" OR "severe colitis" OR "rescue therapy" OR "rescue treatment" OR "medical rescue" OR "salvage therapy" OR "salvage treatment" OR "sequential rescue" OR "second-line rescue" OR "second line rescue" OR "Truelove and Witts" OR "Oxford criteria")  AND  (infliximab OR Remicade OR Remsima OR Inflectra OR "CT-P13" OR cyclosporine OR cyclosporin OR ciclosporin OR ciclosporine OR tacrolimus OR FK506 OR tofacitinib OR Xeljanz OR upadacitinib OR Rinvoq OR filgotinib OR vedolizumab OR ustekinumab OR adalimumab OR golimumab OR "anti-TNF" OR "anti TNF" OR "TNF inhibitor" OR "TNF inhibitors" OR "JAK inhibitor" OR "JAK inhibitors" OR "Janus kinase inhibitor" OR "Janus kinase inhibitors" OR "calcineurin inhibitor" OR "calcineurin inhibitors" OR biologic* OR "biological therapy" OR "biological therapies" OR "small molecule" OR "small molecules" OR "advanced therapy" OR "advanced therapies" OR "accelerated infliximab" OR "accelerated-dose infliximab" OR "accelerated dose infliximab" OR "intensified infliximab" OR "high-dose infliximab" OR "high dose infliximab") |
| Cochrane Central Register of Controlled Trials — CENTRAL | ("ulcerative colitis" OR "ulcerative pancolitis" OR "ulcerative colitides")  AND  ("acute severe ulcerative colitis" OR "acute severe colitis" OR ASUC OR "SR-ASUC" OR "SR ASUC" OR "steroid refractory" OR "steroid-refractory" OR "steroid resistant" OR "steroid-resistant" OR "corticosteroid refractory" OR "corticosteroid-refractory" OR "corticosteroid resistant" OR "corticosteroid-resistant" OR "steroid failure" OR "corticosteroid failure" OR "steroid nonresponse" OR "steroid non-response" OR "corticosteroid nonresponse" OR "corticosteroid non-response" OR "intravenous steroid failure" OR "intravenous corticosteroid failure" OR "fulminant colitis" OR "severe colitis" OR "rescue therapy" OR "rescue treatment" OR "medical rescue" OR "salvage therapy" OR "salvage treatment" OR "sequential rescue" OR "second-line rescue" OR "second line rescue" OR "Truelove and Witts" OR "Oxford criteria")  AND  (infliximab OR Remicade OR Remsima OR Inflectra OR "CT-P13" OR cyclosporine OR cyclosporin OR ciclosporin OR ciclosporine OR tacrolimus OR FK506 OR tofacitinib OR Xeljanz OR upadacitinib OR Rinvoq OR filgotinib OR vedolizumab OR ustekinumab OR adalimumab OR golimumab OR "anti-TNF" OR "anti TNF" OR "TNF inhibitor" OR "TNF inhibitors" OR "JAK inhibitor" OR "JAK inhibitors" OR "Janus kinase inhibitor" OR "Janus kinase inhibitors" OR "calcineurin inhibitor" OR "calcineurin inhibitors" OR biologic OR biological OR "biological therapy" OR "biological therapies" OR "small molecule" OR "small molecules" OR "advanced therapy" OR "advanced therapies" OR "accelerated infliximab" OR "accelerated-dose infliximab" OR "accelerated dose infliximab" OR "intensified infliximab" OR "high-dose infliximab" OR "high dose infliximab") |

*Supplementary Table A2. Reasons for exclusion following full-text assessment*

| **Primary reason for exclusion** | **Reports excluded, n** |
| --- | --- |
| Population did not meet criteria for confirmed steroid-refractory acute severe ulcerative colitis | **22** |
| No eligible comparative treatment group or noncomparative study design | **10** |
| Intervention did not meet the prespecified criteria for eligible medical rescue therapy | **32** |
| No extractable colectomy, colectomy-free survival, or other eligible comparative outcome | **4** |
| Case report, case series, review, editorial, or other ineligible publication type | **6** |
| Duplicate publication, companion report, or substantially overlapping study population | **14** |
| Conference abstract or supplement-only report without an eligible complete peer-reviewed full-text publication | **26** |
| **Total** | **114** |
